# Degradation of IRF6 by TRIM59 in tumor cells triggers PGM1-mediated glycolysis to regulate cell proliferation in neuroblastoma

**DOI:** 10.1038/s41419-025-07932-2

**Published:** 2025-08-12

**Authors:** Liang Zeng, Hui Xu, Meng Li, Liang-Jun Qin, Kai Chen, Feng-Hua Wang, Xiaomin Li, Tianyou Yang, Lei Miao, Hai-Yun Wang

**Affiliations:** 1 Department of Pathology, Guangzhou Women and Children’s Medical Center, Guangzhou Medical University, Guangdong Provincial Clinical Research Center for Child Health, National Children’s Medical Center for South Central Region, Guangzhou, China; 2 Department of Paediatric Surgery, Guangzhou Women and Children’s Medical Center, Guangzhou Medical University, Guangdong Provincial Clinical Research Center for Child Health, National Children’s Medical Center for South Central Region, Guangzhou, China; 3 Department of Thoracic Surgery, Guangzhou Women and Children’s Medical Center, Guangzhou Medical University, Guangdong Provincial Clinical Research Center for Child Health, National Children’s Medical Center for South Central Region, Guangzhou, China; 4 Department of Respiratory, Guangzhou Women and Children’s Medical Center, Guangzhou Medical University, Guangzhou, China

**Keywords:** Paediatric cancer, Cancer metabolism

## Abstract

Neuroblastoma is the most common extracranial malignancy in children, and patients who develop recurrent or metastatic disease are likely to have a much poorer survival prognosis. Herein, by applying random forest and XGBoost machine-learning techniques, we identified interferon regulatory factor (IRF) 6 as the most crucial gene associated with neuroblastoma patient survival. Low IRF6 expression was further determined to be associated with dismal survival in neuroblastoma patients. IRF6 overexpression inhibited cell proliferation in vitro and in vivo and even weakened glycolytic metabolism and increased maximal respiration in SK-N-BE2 and CHP-212 cells. Mechanistically, RNA sequencing, ChIP, and dual-luciferase reporter assays revealed that IRF6 inhibited PGM1 expression by decreasing the transcriptional activity of promoter 3 of PGM1, and PGM1 overexpression may reverse the inhibitory effects of IRF6 on cell proliferation and glycolysis. Additionally, IRF6 expression was diminished in neuroblastoma due to E3 ligase TRIM59-mediated polyubiquitination, and may reverse the promoting effect of TRIM59 overexpression on cell proliferation and glycolysis. Our work thus provides mechanistic insight into the control of glycolysis-mediated disease progression and opens new avenues for developing therapeutic strategies in neuroblastoma.

## Introduction

Neuroblastoma, the most common extracranial solid cancer in children [1], is characterized by frequent metastasis at diagnosis and a tendency for spontaneous remission of tumor cells in infants [2]. The poor prognosis of high-risk neuroblastoma patients, with a 5-year survival rate of less than 50%, is primarily attributed to the prevalence of recurrent/metastatic disease [3]. The Warburg effect, a major metabolic hallmark in cancer cells, involves preferential glucose conversion to lactate, supporting tumor aggressiveness [4, 5]. Reprogramming glucose metabolism is a promising neuroblastoma therapeutic strategy [6]. Thus, further elucidating the molecular mechanisms driving neuroblastoma progression is essential for identifying novel, more effective, and less toxic precision oncology strategies.

Numerous studies have shown that dysregulated transcription factors represent a distinct class of therapeutic targets that modulate cancer-related gene expression by either inhibiting or enhancing cell differentiation and glycolysis in tumors [7]. The transcription factor family of interferon regulatory factors (IRFs), consisting of IRF1‒IRF9 in humans, governs the transcription of interferon (IFN) and IFN-inducible genes. They also boost the production of cytokines and chemokines, which are widely involved in the regulation of apoptosis and tumorigenesis [8]. Among the IRFs, IRF6 is unique [9] and has not been implicated in any of the pathways regulating the activation and function of other IRFs, such as the innate immune response [10]. Initially, IRF6 was reported to regulate craniofacial and epidermal development and maintenance, as seen in Van der Wodude syndrome [10]. Recent studies have demonstrated that IRF6 is downregulated in poorly differentiated squamous cell carcinoma and highly metastatic nasopharyngeal carcinoma, where it promotes invasive behavior [9, 11, 12]. Conversely, in renal clear cell carcinoma and glioma, IRF6 overexpression reduces xenograft growth and prolongs patient survival [13, 14]. This evidence indicates that IRF6 may possess tumor-suppressive properties. However, the role of IRF6 in neuroblastoma is yet to be fully elucidated.

In the present study, we hypothesized that IRF6 acts as a tumor suppressor in neuroblastoma. Thus, we performed a series of experiments to explore the mechanism underlying this function. The results provided evidence that IRF6 expression was low in high-risk neuroblastoma patients and was related to poor survival. IRF6 overexpression inhibits tumor cell growth in vivo and in vitro by attenuating glycolysis. Interestingly, IRF6 targets the PGM1 promoter region and weakens its transcriptional activity in neuroblastoma. A functional study revealed that PGM1 overexpression counteracts the inhibitory effects of IRF6 overexpression on glycolysis-mediated tumor cell growth. Additionally, we found that TRIM59 ubiquitinated IRF6 and promoted its degradation. Our study highlights the essential role of the TRIM59-IRF6-PGM1 axis in regulating glycolysis-mediated neuroblastoma tumor cell growth, providing potential intervention targets for novel therapeutic regimens involving neuroblastoma metabolism.

## Materials and Methods

### Clinical tissue samples

A total of 126 archived formalin-fixed, paraffin-embedded (FFPE) tissue samples were obtained from Guangzhou Women and Children’s Medical Center between October 2018 and October 2022 (Guangzhou, China). Additionally, eight freshly frozen neuroblastoma tissues (low-risk *vs*. high-risk) for the analysis of IRF6 expression were collected and immediately frozen and preserved in liquid nitrogen after surgical resection. All diagnoses were confirmed by two experienced pathologists (L.Z. and H.X.). Written informed consent was obtained from the legal guardians/parents of all neuroblastoma patients, typically upon admission to the hospital. This study was approved by the Institutional Ethics Board in accordance with the Declaration of Helsinki (no. [2024]094A01).

### Dataset processing

The datasets used herein are as follows: The Gene Expression Omnibus (GEO) DataSets portal under accession number GSE16476, and the Cangelosi786 dataset developed in a previous study [15], which can be found under the menu ‘select a dataset for analysis’ on the R2 platform (http://r2.amc.nl). All gene expression values were log2 transformed for subsequent analyses, including screening for IRF1‒9 and survival analysis.

The original RNA-seq data, GTEx, and TARGET153 datasets were re-analyzed using the UCSC TOIL pipeline via the UCSC Xena project. The pipeline included: FastQC (v0.11.5) for quality assessment, CutAdapt (v1.9) for quality control and adapter removal, STAR (v2.4.2a) for sequence alignment, and RSEM (v1.2.25) for gene expression quantification. Count values were transformed using log2 (count + 1) for further analysis. Next, the R package limma (v3.56.2) was used for comparison analysis. Gene expression counts were normalized using counts per million (CPM), retaining genes with CPM > 0.2 in at least three samples. TMM normalization and *voom* weighting were applied, and a linear model was fitted using *ImFit*. After filtering, 21,434 genes remained for comparison analysis.

### The IRFs screening strategy

To evaluate the effect of IRF1‒IRF9 on survival outcomes, two advanced machine-learning algorithms were employed to score the importance of variables based on the DataSet GSE16476. The R package ‘*randomForestSRC*’ (version 3.2.3) was utilized to implement a random forest consisting of survival trees, which effectively handled censored data, with variable importance being assessed with the ‘*VIMP*’ parameter. The ‘*xgboost*’ R package (version 1.7.6.1) was used to apply the XGBoost algorithm, a gradient boosting system that sequentially builds decision trees, with the ‘*gain*’ parameter to measure the importance of variables.

### Cell culture and treatment

Human neuroblastoma cell lines CHP-212, SH-SY5Y, SK-N-BE2, SK-N-SH, and human embryonic kidney 293T (HEK-293T) cells were sourced from the American Type Culture Collection (ATCC, Manassas, VA, USA) and maintained in DMEM medium (GIBCO, NY, USA) supplemented with 10% fetal bovine serum (FBS, GIBCO). All cells were incubated at 37 °C with 5% CO_2_. Short-tandem repeat analysis confirmed the authenticity of all cells, which are also free of mycoplasma contamination.

For treatments, transfected cells were maintained in medium containing the following: 150 μM the glucose analog 2-deoxyglucose (2DG; AbMole, Houston, USA) for the determined experiments; 50 μg/ml the protein synthesis inhibitor CHX (AbMole) for the indicated time (0, 3, 6, and 9 h); 25 μM the proteasome inhibitor MG132 (AbMole) for 9 h or 4 μM the lysosome inhibitor CQ (MCE, Shanghai, China) for 25 h. After treatment, cells were collected, and total protein was extracted using RIPA lysis buffer (Servicebio, Wuhan, China) for further experiments.

### Plasmid construction

The IRF6 coding sequence (CDS) was tagged with Flag, cloned, and inserted into the empty plasmid 3FLAG-EF1a-firefly-luciferase-SV40 to obtain the overexpression plasmid IRF6-3FLAG-EF1a-luciferase-SV40 (GeneChem, Shanghai, China). The TRIM59 CDS, tagged with or without HA, and the PGM1 CDS were cloned and inserted into the empty plasmid pcDNA3.1 (Addgene, MA, USA) to obtain the respective overexpression plasmids. The Myc-IRF6 fusion sequence, featuring the Myc tag sequence added to the N-terminus of the IRF6 CDS region, was cloned and then inserted into the pET28a plasmid (11905ES03, Yeasen, Shanghai, China). IRF6 and TRIM59 siRNA sequences #1, #2, and #3, featuring 3’dTdT overhangs, were designed using the BLOCK-iT™ RNAi Designer. All sequences are listed in Supplementary Table 1.

### Myc affinity isolation

A 5 ml sample of E. coli overexpressing the pET28a-Myc-IRF6 protein was harvested through centrifugation at 12,000 *g* for 1 min to remove the supernatant. The bacteria pellets were resuspended in 1000 μl of lysis buffer containing inhibitors (P2183S, Beyotime, Shanghai, China) and gently mixed, following by lysis on ice for 10 min. Magnetic beads (P2183S, Beyotime) were added to the lysate at a ratio of 20 μl of bead suspension per 500 μl of protein sample. The mixture was incubated on a side-to-side shaker at room temperature for 2 h to allow the Myc-IRF6 fusion proteins to bind to the beads. The beads were subsequently washed three time with lysis buffer containing inhibitors. The bound proteins were eluted by mixing with acid elution buffer (P2183S, Beyotime) at room temperature for 5 min. After separating the beads using a magnetic rack for 10 seconds, the supernatant containing the eluted protein was transferred to a new tube and neutralized by adding 10 μl of neutralization buffer (P2183S, Beyotime). The eluted and neutralized Myc-IRF6 protein was stored at 4 °C for short-term use. The protein concentration was determined using a Nano-600 protein analyzer (Jiapeng Tech., Shanghai, China).

### Cell transfection

Overexpression plasmids or siRNAs were transfected into SK-N-BE2, CHP-212, SH-SY5Y and/or SK-N-SH cells using Lipo2000 transfection reagent (Thermo Fisher Scientific, Waltham, MA, USA) according to the manufacturer’s instructions, and the cells were harvested for further experiments after 24–48 h of transfection. For stable transfection, a lentiviral vector and its negative control were constructed and cotransfected with the IRF6 overexpression plasmid or empty control plasmid into HEK293T cells for 48 h to obtain viral supernatant (GeneChem). The viral supernatant was subsequently used to infect neuroblastoma cells at a multiplicity of infection (MOI) of 10, 20, or 50 for 48 h. Cells were then selected by incubation with conditioned medium containing puromycin (0.5–5 μg/mL; Coolaber, Beijing, China). IRF6 expression in positive neuroblastoma cell clones was confirmed by real-time quantitative PCR (RT-qPCR) and western blot analyses.

### RNA isolation and RT-qPCR

RNA was extracted using TRI reagent (MRC, Inc., Cincinnati, OH, USA), with chloroform for phase separation, isopropanol for RNA precipitation, and 75% ethanol for purification. cDNA was synthesized using random primers and M-MLV reverse transcriptase (Promega, Madison, WI, USA) according to the manufacturer’s instructions. RT**‒**qPCR was performed using GoTaq® qPCR Master Mix (Promega) and a CFX96 Real-time Quantitative PCR System (Bio-Rad, CA, USA). β-catenin was used as an endogenous control. All primers used are listed in Supplementary Table 1.

### Western blot

Freshly frozen tissue samples were homogenized using a cryogenic homogenizer (Servicebio) and lysed on ice for 30 min prior to centrifugation at 12,000 rpm for 10 min at 4 ^o^C to obtain proteins. Cell lysates from the cell lines were centrifuged for 10 min at 12,000 rpm and 4 ^o^C. Equal amounts (20–50 μg) of harvested total proteins were then subjected to SDS-PAGE prior to being transferred to PVDF membranes (Servicebio). The PVDF membranes were subsequently blocked with a 5% skim milk solution and incubated overnight at 4 ^o^C with the appropriate primary antibodies. The targeted proteins were then detected using goat anti-mouse GAPDH (KC-5G5, Kangchen, Shanghai, China) at room temperature for 1 h and visualized using ECL Blotting Substrates (Servicebio). The antibodies used are listed in Supplementary Table 2. Unprocessed images of the immunoblots are shown in Supplementary Fig. 6.

### Immunohistochemistry (IHC)

Briefly, pathological serial sections with a thickness of 4 µm were sliced from the FFPE tumor blocks and used for IHC staining, and the expression of the corresponding proteins was scored as previously described [16, 17]. The IHC assay was conducted using a BOND Polymer Refine Detection (mouse/rabbit IgG) commercial IHC kit (Leica) in accordance with the manufacturer’s instructions. The primary antibodies used are listed in Supplementary Table 2.

### Cell viability assay

Cells were seeded in each well of a 96-well plate, and 20 μl of Cell Counting Kit-8 (CCK-8) reagent (Bioscience, Shanghai, China) was added to each well of the 96-well plate. Finally, the absorbance value of each well at 450 nm was measured once per day for 4 days using a Multiskan™ FC microplate reader (Thermo Fisher Scientific) after 2‒4 h of incubation at 37 °C.

### EdU staining

The cells were seeded into each well of a 6-well plate with the corresponding concentration of EdU reagent for 2 h. The cells were washed twice with PBS for 5 min prior to incubation with 4% paraformaldehyde for 30 min. Finally, the cells were permeabilized with 0.3% Triton X-100 in PBS, and cell proliferation was measured using the BeyoClick™ EdU Cell Proliferation Kit (Beyotime) according to the manufacturer’s instructions. Images at 20× magnification were obtained with an Olympus microscope.

### Flow cytometry

The cells were digested, centrifuged at 1500 rpm for 2 min and washed twice with PBS. The cells were then stained with Annexin V/PI (Bioscience) in the dark at 37 ^o^C for 30 min. The cells were assessed using a BD FACSCalibur™ flow cytometer (BD Biosciences, San Jose, CA, USA), and the apoptosis rate was determined using FlowJo software.

### Glucose uptake

The indicated cells (5 × 10^6^) were suspended in 1 mL of distilled water and then lysed by ultrasonic disruption on ice, followed by incubation in a water bath at 95 °C for 10 min, cooled to room temperature, and then centrifugation at 8000 × *g* at room temperature for 10 min. The resulting supernatant was assessed using a D-Glucose Content Assay (Boxbio, Beijing, China) following the manufacturer’s instructions. An optical absorption–concentration standard curve was established to calibrate the glucose concentration.

### Lactate production

The lactic acid (LA) concentrations were measured using an LA Content Assay (Boxbio). In brief, cells were suspended in extraction solution A and lysed by ultrasonic disruption on ice, followed by centrifugation at 12,000 × g at 4 °C for 10 min. A total of 800 μl of the supernatant was mixed thoroughly with 150 μl of extraction solution B, followed by centrifugation at 12,000 × *g* at 4 °C for 10 min. The final supernatant was measured according to the manufacturer’s instructions, and lactate concentration was calibrated using an optical absorbance‒concentration standard curve.

### Extracellular acidification rate (ECAR) and oxygen consumption rate (OCR)

The ECAR and OCR were assessed using the Seahorse XF glycolysis stress test kit and Seahorse XF Cell Mito Stress Test Kit (Agilent Technologies, Palo Alto, CA, USA), respectively. Measurements were performed using the Seahorse XFe24 Analyzer (Agilent Technologies, kindly provided by the Zhongshan School of Medicine, Sun Yat-sen University). SK-N-BE2 and CHP-212 cells (20,000 cells/well) were plated in 24-well cell culture microplates (XFe24 FlµxPak) in 500 µl of medium and incubated overnight at 37 °C under 5% CO_2_. An XF24 sensor cartridge (Agilent Technologies) was hydrated overnight at 37 °C under 5% CO_2_.

For ECAR measurement, cells were cultured in XF medium (without glucose, supplemented with 2 mM glutamine, pH = 7.4) and incubated at 37 °C in a non-CO_2_ incubator to maintain glucose starvation conditions. ECAR was measured under glucose starvation conditions (0–18 min, non-glycolysis). At 18 min, 56 μl of glucose (10 mM final concentration) was added to induce glycolysis, and ECAR was measured at 24, 30, and 36 min. At 36 min, 62 μl of oligomycin was injected to evaluate glycolysis capacity, leading to increased ECAR at 42, 48, and 54 min. Finally, 69 μl of 2DG was added at 54 min to competitively inhibit total glycolysis, with measurement taken at 60, 66, and 72 min.

For OCR measurements, cells were cultured in XF medium (supplemented with 2 mM glutamine, pH = 7.4) and incubated at 37 °C for 1 h in a non-CO_2_ incubator to maintain glucose starvation conditions. Basal OCR was measured at 10, 20, and 30 min. At 30 min, 56 μl of oligomycin (10 mM final concentration) was added to measure ATP production, with OCR measurement at 40, 50, and 60 min. At 60 min, 62 μl of FCCP was subsequently injected to evaluate maximal respiration, with OCR measured at 70, 80, and 90 min. Finally, 69 μl of a rotenone and antimycin A mixture was injected at 90 min to measure non-mitochondrial respiration, with OCR measured at 100, 110, and 120 min.

Data were analyzed using Seahorse XF24 Wave software, with ECAR reported in mpH/min and OCR in pmol/min.

### Co-IP assay

Cells were lysed with IP lysis buffer (Servicebio) containing a protease inhibitor cocktail (Servicebio), blended repeatedly with a pipette for 30 min at 4 °C and then centrifuged at 12,000 rpm at 4 °C for 10 min to obtain the protein-containing supernatant. This supernatant was subsequently incubated with 2.0 μg of anti-Flag-tag antibody (CST) or anti-IgG antibody (Proteintech) overnight at 4 °C. The antibody-supernatant mixture was incubated with Pierce Protein A/G Magnetic Beads (Millipore) for bead capture at room temperature for 2 h. The beads were then washed four times with IP lysis buffer, and 80 μl of protein loading buffer was added for protein denaturation at 100 °C for 10 min. Finally, 10 μl of proteins was separated by SDS‒PAGE and subjected to western blot analysis.

For the IP ubiquitination assay, MG132-treated SK-N-BE2 and CHP-212 cells were collected. For the exogenous ubiquitination assay, HEK293T cells were co-transfected with His-UB (#107392, Addgene), Flag-IRF6, and/or HA-TRIM59 plasmids and pre-treated with or without 25 μM MG-132. The immunoprecipitated complexes were detected by western blot analysis using an anti-ubiquitin antibody (E4I2J, CST), according to the manufacturer’s protocol.

The in vitro ubiquitination assay was performed as previously described with minor modifications [18]. The 30 μl reaction mixture contained the following components: 50 ng of UBA1/UBE1 (UBE-024, UB-biotech, Changchun, China), 200 ng of UBE2D1 (11432-H07E, Sino Biological, Beijing, China), 500 ng of purified IRF6, 5 μg of ubiquitin (RPA164Mi01, Cloud-Clone Corp., Wuhan, China), and 1.5 μl 20x reaction buffer (1 M Tris, pH7.5, 40 mM ATP [M9905, AbMole], 100 mM MgCl_2_, 40 mM DTT). The reaction was initiated by adding GST-tagged TRIM59 (Ag29560, Proteintech). The mixture was incubated at 30 °C for 1.5 h with rotation and terminated by adding 2 x loading buffer. Subsequently, western blot analysis was performed to evaluate the ubiquitination levels.

### RNA sequencing and bioinformatics analysis

RNA sequencing was performed on total RNA from SK-N-BE2-Vector and SK-N-BE2-IRF6 cells, including RNA isolation, library construction, and 150 bp paired-end sequencing, in triplicate for each group on an Illumina sequencing platform (BerryGenomics, Beijing, China). Gene expression levels were quantified using RSEM (version 1.3.1) and then annotated with the GRCh38 reference genome and the Genecode v44 transcript. Simultaneously, transcript-level counts were normalized to transcripts per million (TPM). Differential gene expression (DEG) analysis was performed using the voom algorithm, with significant DEGs identified based on a Benjamini & Hochberg adjusted *p*-value < 0.05, and a |log2-fold change|> 1. Gene ontology (GO) enrichment analysis was conducted using the R package *clusterProfiler* (version 4.10.0), with GO terms having an adjusted *p*-value less than 0.05 considered significantly enriched.

### Chromatin immunoprecipitation (ChIP) -qPCR

ChIP‒qPCR was performed using a ChIP kit (Bersinbio, Guangzhou, China) according to the manufacturer’s instructions. Briefly, cells were fixed with 1% formaldehyde, washed, and collected by centrifugation (1000 × *g* for 5 min at 4 °C). The pellet was resuspended in lysis buffer supplemented with 1% protease inhibitors and dithiothreitol, homogenized, incubated on ice for 10 min, and sonicated. The samples were centrifuged (13,000 × *g* for 10 min at 4 °C), and the shared chromatin was used as input and incubated with an anti-Flag antibody. Rabbit IgG (Bersinbio) was used as the isotype control. After precipitation with Pierce Protein A/G Magnetic Beads and digestion of RNA and protein, DNA was purified using GoTaq® qPCR Master Mix according to the manufacturer’s instructions. The qPCR primers used to evaluate the promoter regions of PGM1 are listed in Supplementary Table 1.

### Dual-luciferase reporter assay

Potential PGM1 binding sites in IRF6 were predicted using the bioinformatics tool JASPAR (2024 v). The IRF6 gene with a potential PGM1 binding site was inserted into the pGL3-Basic plasmid vector to obtain the reporter vector pGL3-Basic-PGM1-promoter-wild (Jennio, Guangzhou, China). To mutate the putative binding site of PGM1, the sequence of the putative binding site was replaced, as indicated, to form the pGL3-Basic-PGM1-promoter-mut (Jennio). Relative luciferase activity was measured 48 h after transfection, and firefly luciferase activity was normalized to Renilla luciferase activity (GeneCopoeia, MD, USA).

### Animal experiments

The SK-N-BE2-IRF6-overexpression and SK-N-BE2-vector cells (5.0 × 10^6^/120 μl per mouse) were injected into the right back axillary dermis of 4-week-old male NCG mice (GemPharmatech, Nanjing, China). This model was designed to detect subcutaneous tumorigenesis. Tumor length (L) and width (W) were measured using a Vernier caliper. Tumor volume was calculated using the equation V = 1/2 × L × W^2^. Approximately four weeks later, the mice were sacrificed by carbon dioxide asphyxiation. The tumors were excised and fixed in 4% formalin. After paraffin embedding and sectioning, the sections were used for further experiments. All animal experimental procedures were performed in accordance with the guidelines of the Institutional Animal Care and Use Committee of Guangzhou Jennio (JENNIO-IACUC-2024-A032).

### Statistical analysis

Data are presented as the mean ± standard deviation (SD) of three independent experiments. Survival curves were plotted using the Kaplan‒Meier method, and independent prognostic factors were analyzed using univariate and multivariate Cox regression. Differences between two groups were analyzed by two-tailed unpaired Student’s *t*-test or the chi-square test, and differences in survival were analyzed by the log-rank test. The relative relationships between the expression of IRF6, TRIM59, PGM1, and other genes were determined using Pearson’s correlation coefficient *(r)*. All statistical analyses were performed using GraphPad Prism or the R software (version 4.2.3). A *p*-value of less than 0.05 was considered statistically significant.

## Results

### Low IRF6 expression is associated with a dismal prognosis in neuroblastoma patients

**Fig. 1:**
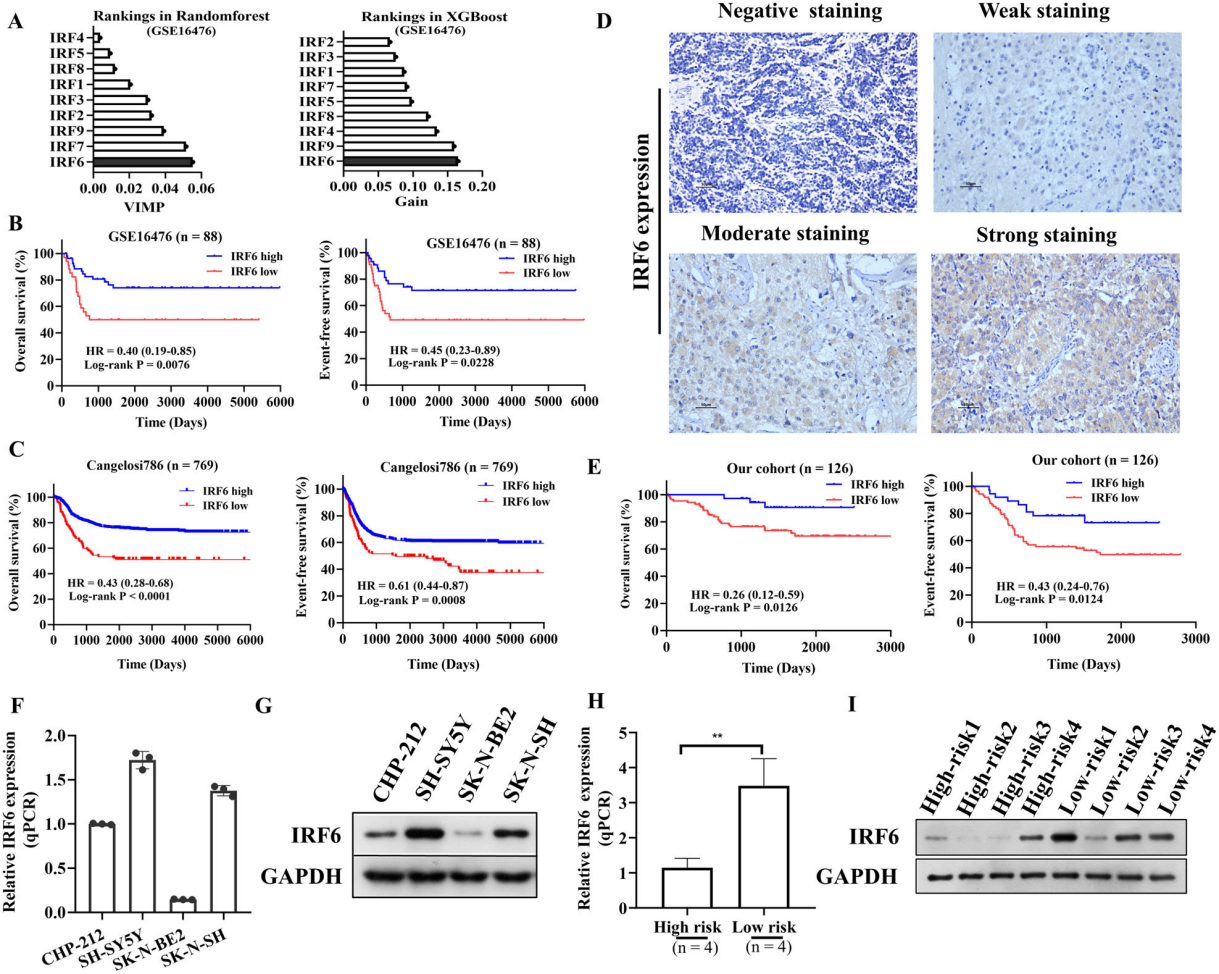
IRF6 is downregulated and correlated with poor survival in neuroblastoma patients. **A** Two advanced machine learning methods, random forest and XGBoost, are depicted in bars for the comparison of importance scores of the IRF1-9 genes, highlighting the potential importance of IRF6 in the pathogenesis and prognosis of neuroblastoma. **B**, **C** Prognostic significance of IRF6 in 88 and 769 patients with neuroblastoma (GSE16476 and Cangelosi786), as assessed by Kaplan–Meier analysis. **D** Representative images of IHC staining of IRF6 in neuroblastoma tissues. Scale bars: 50 μm. **E** Kaplan–Meier analysis of overall survival and event-free survival based on IRF6 expression in 126 patients with neuroblastoma. **F**, **G** RT‒qPCR and western blot analyses of IRF6 expression in 4 neuroblastoma cell lines (CHP-212, SH-SY5Y, SK-N-BE2, and SK-N-SH). **H**, **I** RT‒qPCR and western blot analyses of IRF6 expression in neuroblastoma tissues from high-risk and low-risk patients. In **F**, the data are presented as the means ± SDs of three independent experiments, and the *p* values were determined by two-tailed Student’s *t* test; and in **B**, **C** and **E**, the *p* values were determined by the log-rank test. IRF6 interferon regulatory factor 6.

Given the significant role of several IRFs in the regulation of tumorigenesis, we aimed to evaluate their prognostic significance in neuroblastoma patients. Initially, we used the forest random and XGBoost algorithms to screen IRFs based on the public dataset GSE16476, assessing their importance using VIMP and Gain criteria (Supplementary Fig. 1A, B). IRF6 emerged as the most important gene, with the highest score across both algorithmic approaches (Fig. 1A). Since IRF6 has not yet been previously studied in neuroblastoma, we selected it for further investigation. Kaplan‒Meier survival analysis revealed that low IRF6 expression was associated with a poor overall and event-free survival in neuroblastoma patients, based on the GSE16476 and Cangelosi786 datasets (Fig. 1B, C). To further determine the clinical significance of the IRF6 protein in neuroblastoma, we performed IHC staining with an antibody against IRF6, which showed positive staining in the cytoplasm, in a cohort of 126 neuroblastoma tissues (Fig. 1D). Patients were divided into high- or low-IRF6 expression groups (Supplementary Table 3), and Kaplan‒Meier analysis similarly revealed that neuroblastoma patients with low IRF6 expression had worse overall and event-free survival than those with high IRF6 expression (Fig. 1E). In addition, multivariate Cox analysis confirmed IRF6 remained an independent prognostic factor, even when considering age, MYCN status, COG risk stratification, and INSS (Supplementary Fig. 1C, D).

We subsequently analyzed IRF6 expression and found it to be consistently downregulated in neuroblastoma tissues compared to that in normal adrenal gland tissues (Supplementary Fig. 1E, F). These findings indicate that IRF6 may be more highly expressed in low-risk neuroblastoma patients, pointing to its potential anticancer role. More importantly, we utilized RT‒qPCR and western blotting to measure IRF6 expression in fresh-frozen tissues from 4 with high-risk neuroblastoma patients and 4 with low-risk neuroblastoma patients, as well as in the intermediate-risk cell line SH-SY5Y and the low-risk cell line SK-N-SH, and the high-risk cell lines CHP-212 and SK-N-BE2 [19]. These results confirmed that the mRNA and protein expression levels of IRF6 were significantly lower in high-risk cell lines (Fig. 1F, G) and high-risk neuroblastoma tissues (Fig. 1H, I). Collectively, these results suggest that IRF6 is downregulated in high-risk neuroblastoma patients and correlated with poor survival.

### IRF6 inhibits glycolysis-mediated neuroblastoma cell proliferation

**Fig. 2:**
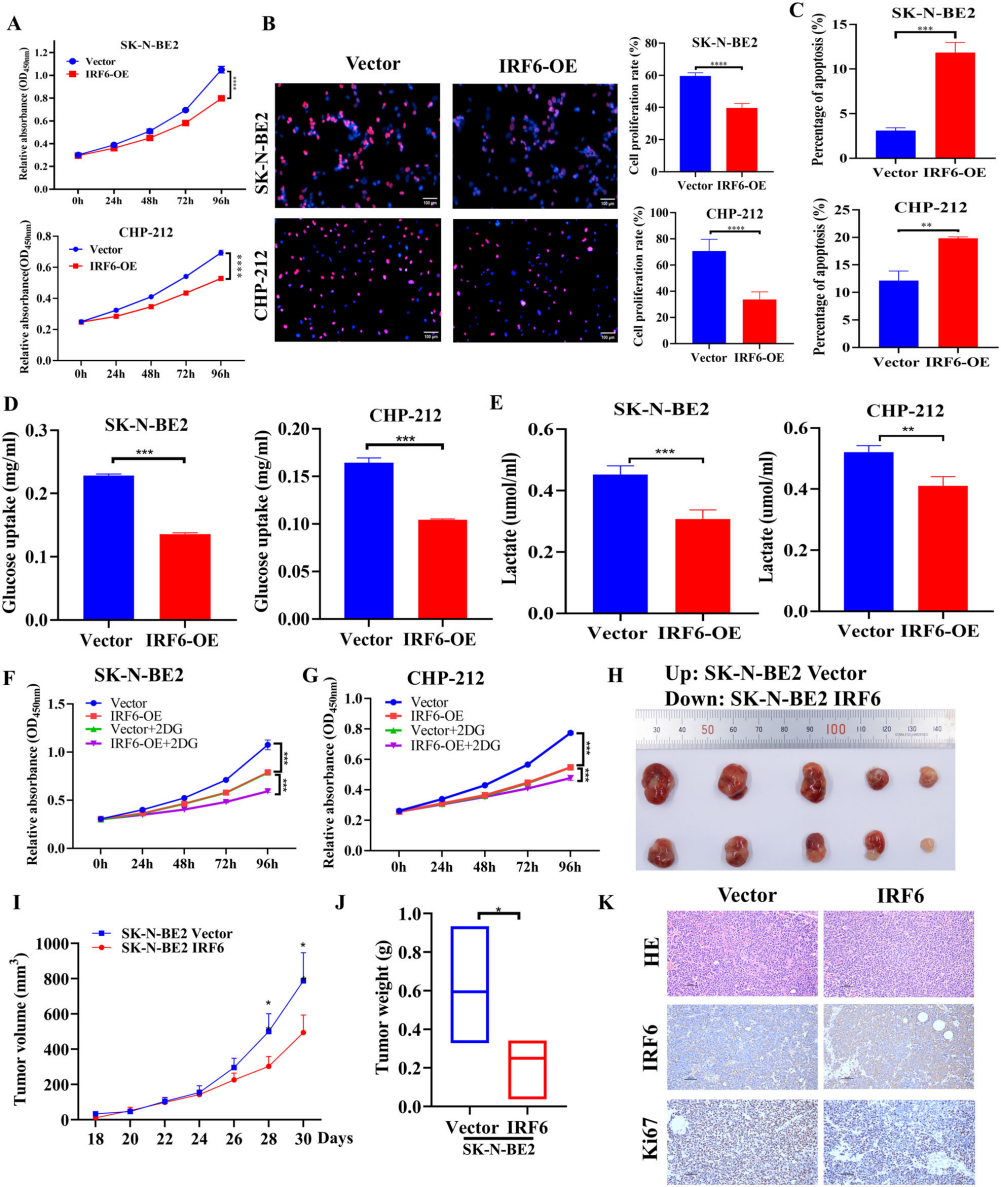
IRF6 suppresses glycolysis-mediated neuroblastoma cell proliferation. After transfecting SK-N-BE2 and CHP-212 cells with Flag-IRF6 or the corresponding empty vector, cell proliferation and viability were evaluated using a CCK-8 assay (**A**), an EdU assay (**B**), and apoptosis was assessed using flow cytometry (**C**). Glucose uptake (**D**) and lactate production (**E**) were also evaluated. After SK-N-BE2 and CHP-212 cells were transfected with Flag-IRF6 or the corresponding empty vector, cell proliferation abilities were assessed using the CCK-8 assay under the condition of 2DG treatment in the indicated cell groups (**F**, **G**). SK-N-BE2 cells stably expressing Flag-IRF6 or empty vector were injected subcutaneously into NCG mice to establish xenograft models. Macroscopic images (**H**), volumes (**I**) and weights (**J**) of excised tumors in each group (*n* = 5). **K** Representative images of HE and IHC staining results with IRF6 and Ki67 in excised mouse tumors from each group; Scale bar: 50 μm. The data in **A**–**G** are presented as the means ± SDs of three independent experiments, and the *p* values were determined by two-tailed Student’s *t* test.

To precisely demonstrate the effect of IRF6 on tumorigenesis, we stably transfected CHP-212 and SK-N-BE2 cells with an IRF6-overexpressing plasmid or empty vector (Supplementary Fig. 2A–D). As shown in Fig. 2A, B, the overexpression of IRF6 markedly decreased the cell viability and proliferation rate of the indicated neuroblastoma cells. Consistent with these findings, the apoptosis rate was dramatically increased in IRF6-overexpressing neuroblastoma cells (Fig. 2C, Supplementary Fig. 2E). Conversely, IRF6 knockdown in neuroblastoma cell lines SH-SY5Y and SK-N-SH using IRF6 siRNAs or a control plasmid (Supplementary Fig. 2F, G) led to increased cell growth (Supplementary Fig. 2H, I).

Since glucose metabolism is comprehensively involved in the development of cancer in cells and IRF6 was previously implicated in the regulation of glycolysis in other neural cancers, such as glioma [13], we next tested whether IRF6 modulates the glycolytic phenotype in neuroblastoma cells. The process of glycolysis can be stimulated by elevated glucose intake, resulting in the production of lactate, and our findings indicated that IRF6 overexpression decreased both glucose uptake and lactate secretion (Fig. 2D, E), while IRF6 knockdown increased both (Supplementary Fig. 2J, K). More importantly, the ECAR and OCR, which are specific and classic indicators for glycolytic acidification and oxygen consumption, were simultaneously measured using a Seahorse XF analyzer. Not surprisingly, we observed a lower level of glycolysis and glycolytic capacity cells and a greater level of maximal respiration and ATP production in both SK-N-BE2 and CHP-212 cells overexpressing IRF6 than in control cells (data shown in Figs. 4H, I, 6E, F). Furthermore, we used the glucose analog 2DG to block glycolysis to determine the effects of 2DG on glycolysis-mediated cell proliferation. As shown in Fig. 2F, G, cell viability was significantly lower in the IRF6-overexpressing group treated with 2DG than that in the corresponding group without 2DG treatment in the SK-N-BE2 and CHP-212 cells.

To further confirm that IRF6 regulates the growth of neuroblastoma cells in vivo, we established a tumor-bearing model by the subcutaneous injection of SK-N-BE2 cells with stable overexpression of IRF6 into NCG mice (*n* = 5 per group). Compared with those in the vector group, the macroscopic size, volume, and weight of the tumors in the IRF6-overexpressing group were significantly lower (Fig. 2H–J). The relative levels of IRF6 and Ki67 proteins in the tumor tissues of the indicated mice were subsequently evaluated by IHC. The results revealed that the overexpression of IRF6 decreased the Ki67 expression (Fig. 2K). These findings reveal that IRF6 overexpression attenuated glycolysis-mediated cell proliferation, indicating a crucial role of IRF6 in neuroblastoma.

### IRF6 inhibits the expression of PGM1 by binding to its promoter region

**Fig. 3:**
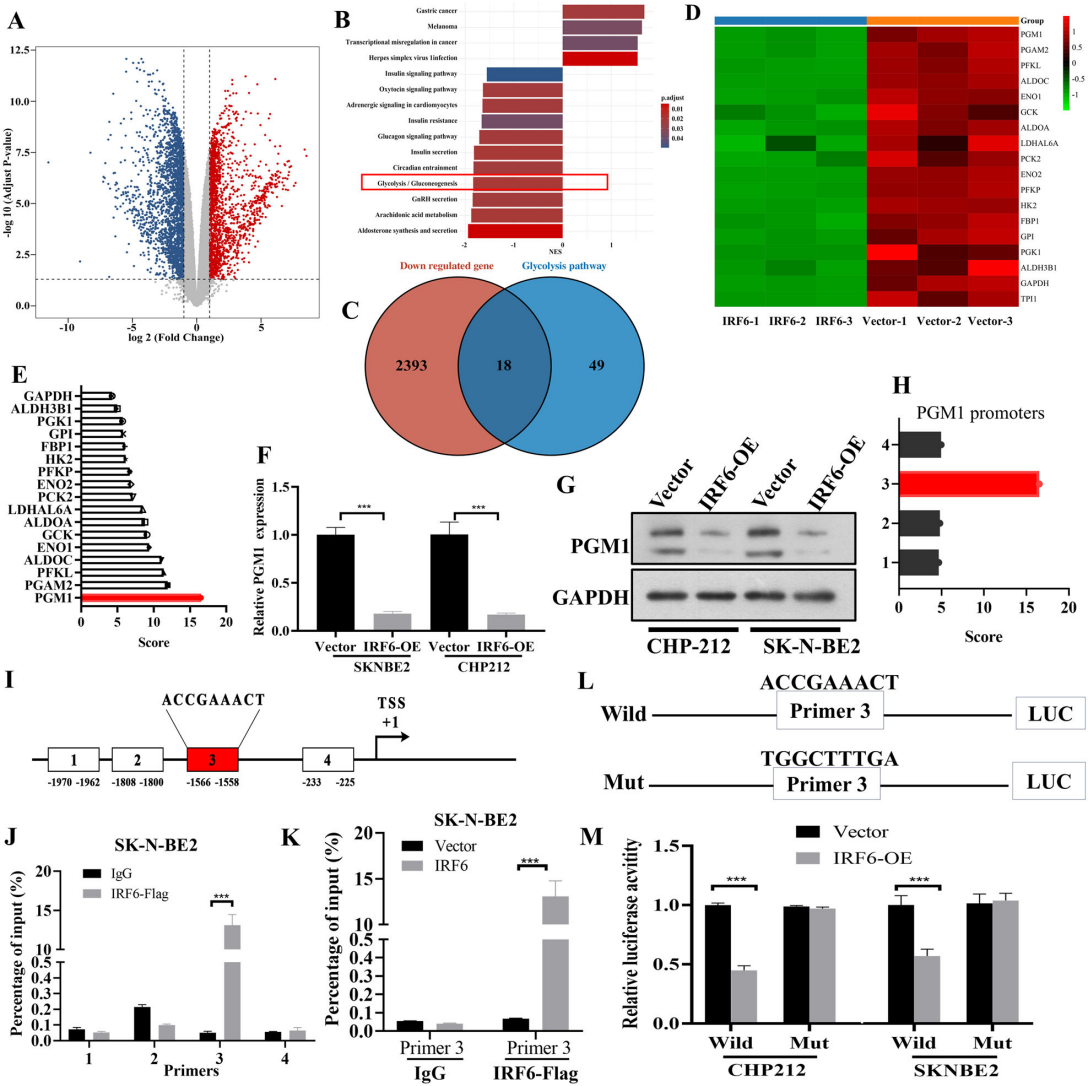
IRF6 binds to the PGM1 promoter and transcriptionally represses PGM1. **A** Volcano plot displaying the DEG distribution between the SK-N-BE2 vector and SK-N-BE2 IRF6 groups. **B** KEGG analysis identified the glycolysis/gluconeogenesis pathways involved in the biological process. **C** Venn diagram showing the numbers of downregulated genes and genes specific for glycolysis pathways. **D** Heatmap displaying the expression of 18 candidate genes shown in (**C**). **E** JASPAR was used to predict the binding scores between IRF6 and the 18 candidate genes. **F**, **G** RT‒qPCR and western blot analyses were used to determine the mRNA and protein expression levels of PGM1, respectively, in the SK-N-BE2 and CHP-212 cells overexpressing IRF6 and the corresponding empty vector. **H** JASPAR was used to predict the binding scores between the IRF6 and PGM1 promoters. **I** Schematic representation of the promoter regions of PGM1. Precipitated DNA was amplified by a PCR assay with primers specific for regions 1‒4 (**J**) or region 3 (**K**). **L**, **M** Transcriptional activities of IRF6 on PGM1 promoter fragments with mut and wild-type sequences as indicated in SK-N-BE2 and CHP-212 cells. The data in **F**, **J**, **K**, **M** are presented as the means ± SDs of three independent experiments, and the *p* values were determined by two-tailed Student’s *t* test. TSS transcriptional start site.

Next, we identified potential target genes of IRF6 that are crucial for its associated functions. SK-N-BE2 cells transfected with an empty vector or an IRF6 overexpression plasmid were subjected to transcriptome sequencing. Notably, DEGs, including downregulated and upregulated genes, were markedly regulated by the overexpression of IRF6 in SK-N-BE2 cells (Fig. 3A). KEGG results revealed that the glycolysis/gluconeogenesis pathways were enriched (Fig. 3B). Therefore, we investigated whether IRF6 overexpression inhibits the expression of downstream targets that attenuate glycolysis in neuroblastoma cells. To identify key genes that IRF6 likely interacts with and are influenced by IRF6 expression in the context of neuroblastoma metabolism, we intersected the downregulated DEGs with those involved in the glycolysis/gluconeogenesis pathways, resulting in a set of 18 genes (Fig. 3C, D). To pinpoint the gene with the most significant impact, we utilized the JASPAR website, which indicated the highest binding affinity between IRF6 and PGM1 (Fig. 3E).

Furthermore, qRT-PCR and western blot analyses revealed that mRNA and protein expression levels of PGM1 were mostly inhibited by the overexpression of IRF6 (Fig. 3F, G). These results indicate that IRF6 might inhibit the expression of PGM1 at the mRNA and protein levels. Similarly, JASPAR predicted that PGM1 promoter 3 was most likely to bind to IRF6 (Fig. 3H, I), and ChIP-qPCR experiments in SK-N-BE2 cells confirmed that IRF6 directly bound to the PGM1 promoter 3 (Fig. 3J, K). Next, we cloned both wild-type and mutant-type sequences of the PGM1 promoter 3 into the pGL4-luciferase reporter vector to investigate the transcriptional functionality of IRF6 binding (Fig. 3L). IRF6 overexpression significantly reduced luciferase activity in cells containing the wild-type PGM1 promoter sequence. By contrast, mutations in the promoter region weakened or even abolished this effect, suggesting that consensus sequences were essential for IRF6 binding and transcriptional regulation (Fig. 3M). These observations suggest that IRF6 directly regulated PGM1 transcription by binding to its promoter.

### PGM1 reverses the glycolysis-mediated tumor cell growth inhibition

**Fig. 4:**
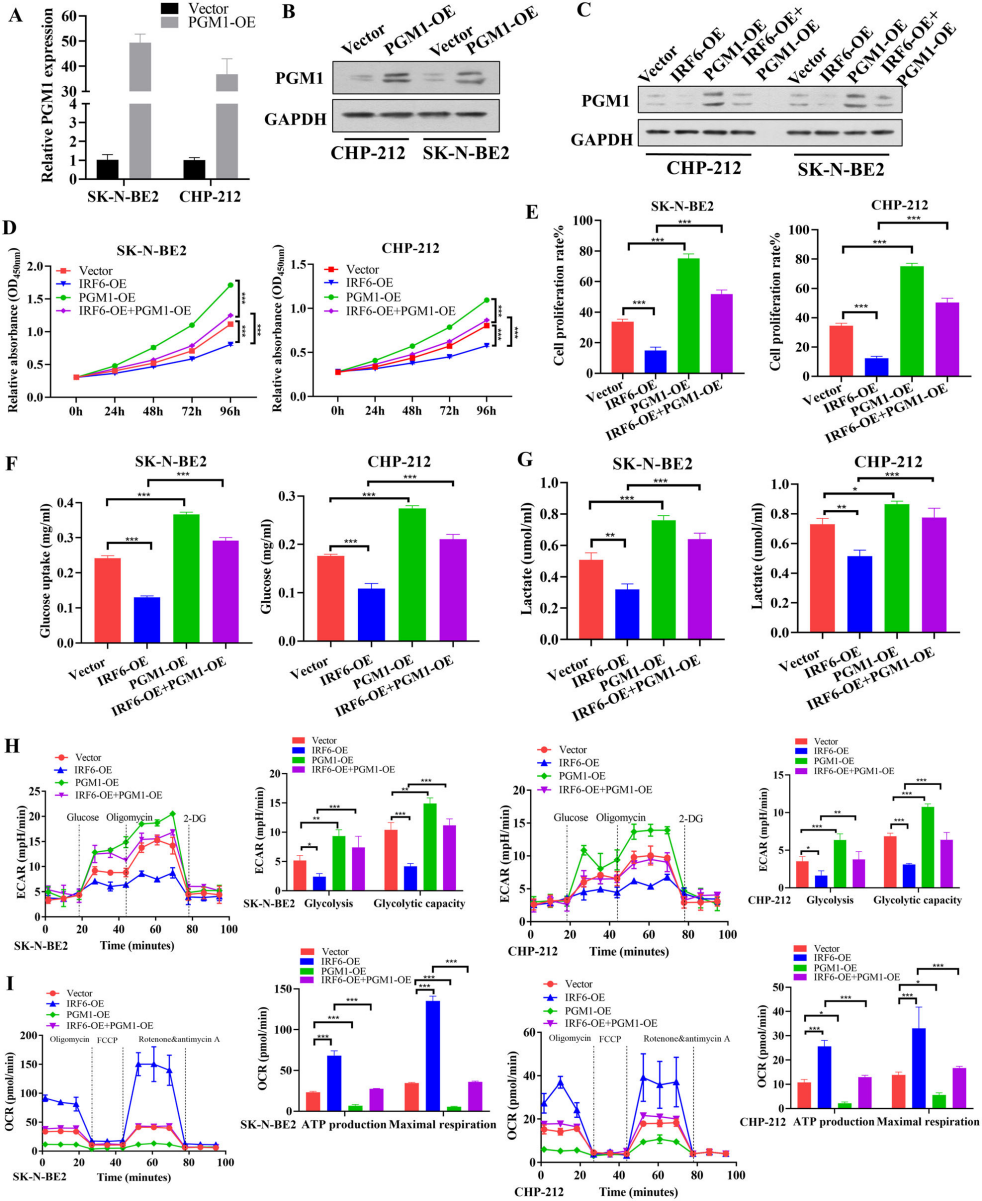
PGM1 reverses the inhibitory effect of IRF6 on glycolysis-mediated tumor cell proliferation. After SK-N-BE2 and CHP-212 cells were transfected with PGM1 or the corresponding empty vector, PGM1 mRNA and protein expression were evaluated by RT‒qPCR (**A**) and western blot (**B**), respectively. Western blotting analysis revealed that PGM1 overexpression reversed the inhibitory effect of IRF6 on PGM1 expression (**C**). SK-N-BE2 and CHP-212 cells were transiently cotransfected with Flag-IRF6 and the corresponding empty vector plus PGM1 or the corresponding control plasmid and were then used for functional experiments. Cell viability (**D**) and proliferation (**E**) were evaluated using CCK-8 and EdU assays, and glucose uptake (**F**), lactate (**G**), ECAR (**H**), and OCR (**I**) were also evaluated. The data in **(A**, **D**–**I**) are presented as the means ± SDs of three independent experiments, and the *p* values were determined using two-tailed Student’s *t* test.

To test whether IRF6 regulates glycolysis-mediated tumor progression by inhibiting PGM1 transcription in neuroblastoma, we constructed a PGM1 overexpression plasmid (Fig. 4A, B) and then cotransfected it with Flag-IRF6 and PGM1 plasmids or corresponding empty vectors for subsequent experiments in SK-N-BE2 and CHP-212 cells. We found that the expression of PGM1 obviously reversed the inhibitory effect of IRF6 overexpression on PGM1 protein expression **(**Fig. 4C). Further functional experiments revealed that PGM1 overexpression rescued the inhibitory effects of IRF6 overexpression on cell viability (Fig. 4D) and the proliferate rate (Fig. 4E, Supplementary Fig. 3A).

Moreover, IRF6 overexpression decreased glucose uptake and lactate production, and these decreases were reversed by ectopic expression of PGM1 in SK-N-BE2 and CHP-212 cells (Fig. 4F, G). Similarly, the inhibitory effects of IRF6 overexpression on glycolysis and glycolytic capacity were reversed by PGM1 overexpression in both SK-N-BE2 and CHP-212 cells (Fig. 4H), and the opposite results were observed in the OCR assay (Fig. 4I). Taken together, these findings suggest that IRF6 overexpression inhibits glycolysis-induced cell proliferation by repressing PGM1 transcription.

### TRIM59 facilitates the degradation of IRF6 by increasing its ubiquitination

**Fig. 5:**
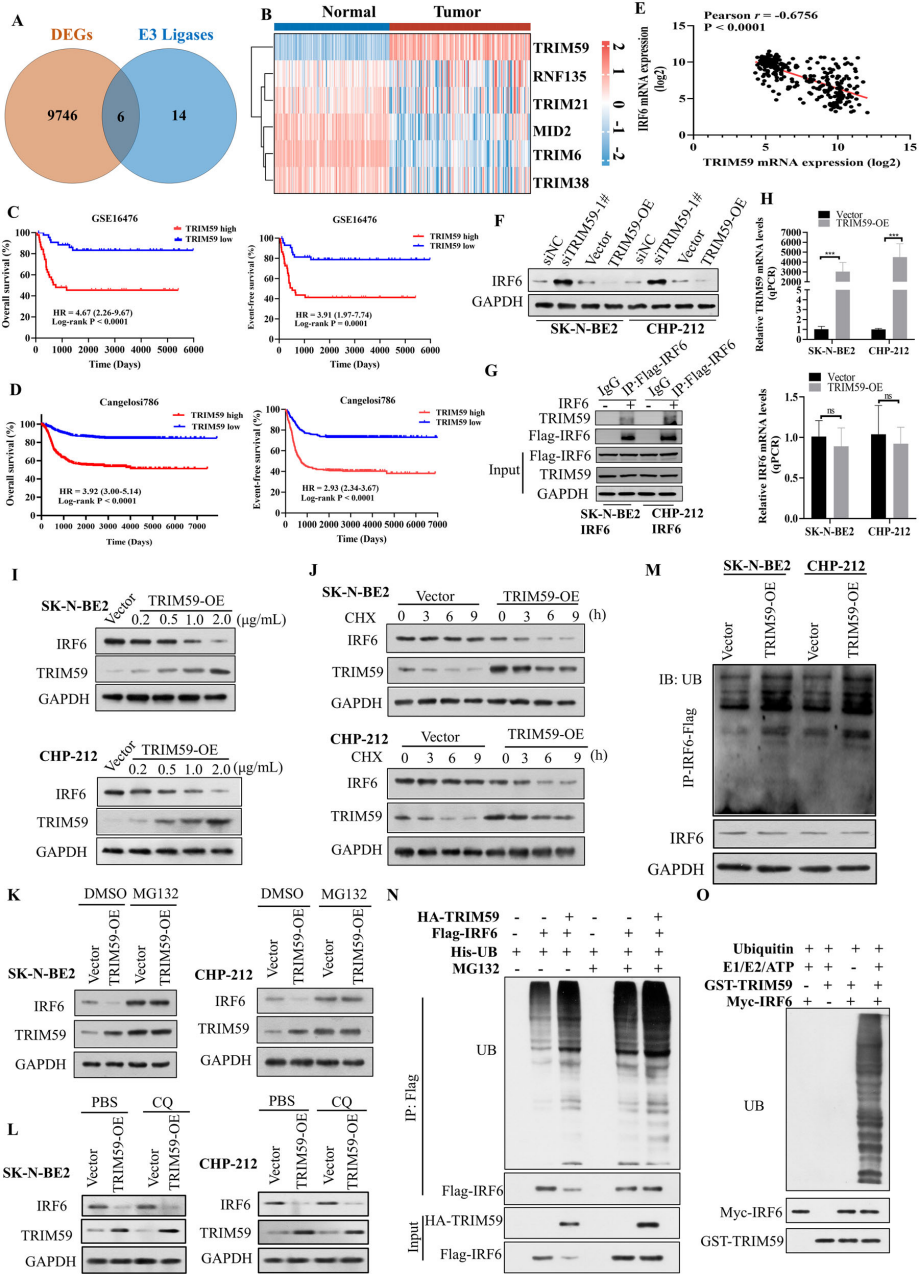
TRIM59 ubiquitinates the IRF6 protein and causes its degradation. **A** Venn diagram showing the number of DEGs genes and E3 ligases. **B** Heatmap displaying the expression of the 6 candidate E3 ligases shown in (**A**). Kaplan–Meier analysis of overall survival and event-free survival based on TRIM59 expression in the GSE16476 (**C**) and Cangelosi786 datasets (**D**) of neuroblastoma patients. **E** The negative correlation between TRIM59 and IRF6 expression was assessed according to the expression of TRIM59 and IRF6 in the GTEx and TARGET 153 datasets. **F** Western blot analysis confirmed the negative correlation between TRIM59 and IRF6 expression in SK-N-BE2 and CHP-212 cells. **G** Co-IP with an anti-Flag antibody and an anti-IgG antibody in SK-N-BE2 and CHP-212 IRF6-overexpressing cells revealed the association of IRF6 and TRIM59. **H**, **I** TRIM59 decreased the protein level of IRF6 in a dose-dependent manner but did not affect its mRNA level in SK-N-BE2 and CHP-212 cells. **J** Effects of CHX treatment on the IRF6 protein level and corresponding grayscale analysis of SK-N-BE2 and CHP-212 cells transfected with TRIM59 or the corresponding empty vector. **K**, **L** Effects of MG132 and CQ treatment on the IRF6 protein level in SK-N-BE2 and CHP-212 cells transfected with TRIM59 or the corresponding empty vector. **M** IP experiments with an anti-Flag-IRF6 antibody were used to measure the ubiquitination levels of IRF6 in transfected SK-N-BE2 and CHP-212 cells treated with MG132. **N** HEK293T cells were co-transfected with plasmids encoding His-UB, HA-TRIM59 and either Flag-IRF6 or empty vector. After 42 h, cells were treated with or without MG132 for 6 h. Proteins were immunoprecipitated with a Flag antibody. Ubiquitination was detected using an anti-ubiquitin antibody, while IRF6 and TRIM59 were detected using anti-Flag and anti-HA antibodies, respectively. **O** Purified GST-TRIM59 was incubated with the indicated reaction components. The reaction mixture was subjected to Western blot using antibodies against ubiquitin, Myc, and GST. The data in **H** is presented as the means ± SDs of three independent experiments, and the *p* values were determined by two-tailed Student’s *t* test; and in **C** and **D**, the *p* values were determined by the log-rank test.

To elucidate the mechanism by which IRF6 expression is kept low, thereby affecting glycolysis-mediated tumorigenesis in neuroblastoma cells, we analyzed the DEGs between neuroblastoma and adrenal gland tissues using the TARGET and GTEx datasets (UCSC Xena). Additionally, given that ubiquitination is a major mechanism promoting protein degradation [20], we hypothesized that specific E3 ligases bind to IRF6, promoting its ubiquitination and degradation in neuroblastoma. Therefore, we used the UbiBrowser website, an integrated bioinformatics platform, to predict potential E3 ligases that may interact with and ubiquitinate IRF6. The predicted E3 ligases were sorted based on confidence scores calculated using the naïve Bayes classifier [21], yielding 20 high-confidence candidates. A Venn diagram and heatmap analysis revealed six E3 ligases with significantly differential expression: TRIM59, RNF135, TRIM21, MID2, TRIM6, and TRIM38 (Fig. 5A, B). Kaplan‒Meier analysis showed that neuroblastoma patients with high TRIM59 expression had worse overall and event-free survival compared to those with low TRIM59 expression (Fig. 5C, D). Survival curves for the other five E3 ligases are shown in Supplementary Fig. 4A, B. Subsequently, correlation analysis revealed a negative correlation between TRIM59 and IRF6, but positive correlations between the other five E3 ligases and IRF6 (Fig. 5E, Supplementary Fig. 4C). Overall, these results led us to hypothesize that the E3 ligase TRIM59 is capable of binding to and ubiquitinating IRF6.

To confirm the effect of TRIM59 on IRF6 expression, we transiently transfected SK-N-BE2 and CHP-212 cells with a TRIM59-overexpressing plasmid, empty vector, TRIM59 siRNAs, or control plasmid (Supplementary Fig. 4D–F). Western blot analysis confirmed a negative correlation between TRIM59 and IRF6 in these cells (Fig. 5F). We performed IP with an anti-Flag antibody or IgG in SK-N-BE2 and CHP-212 cells transfected with the Flag-IRF6 plasmid, followed by western blot analysis, which revealed that IRF6 may bind to the E3 ligase TRIM59 (Fig. 5G). TRIM59 overexpression reduced IRF6 protein levels in a dose-dependent manner but did not affect its mRNA level in SK-N-BE2 and CHP-212 cells (Fig. 5H, I). Consistent with the above results, overexpression of TRIM59 markedly accelerated the degradation of endogenous IRF6 in neuroblastoma cells after treatment with CHX, indicating that TRIM59 could shorten the half-life of the IRF6 protein (Fig. 5J).

To determine whether TRIM59 promotes protein degradation of IRF6 via the ubiquitin‒proteasome pathway or the lysosomal pathway, SK-N-BE2 and CHP-212 cells were transfected with TRIM59 or the corresponding empty vector and then treated with the proteasome inhibitor MG132 or the lysosome inhibitor CQ. We found that TRIM59-mediated destabilization of IRF6 was reversed by MG132 but not by CQ, indicating that TRIM59 promoted IRF6 protein degradation in a ubiquitin‒proteasome‒dependent manner (Fig. 5K, L). Furthermore, we tested the effects of TRIM59 on the ubiquitination of IRF6 and found that the overexpression of TRIM59 markedly promoted the ubiquitination of IRF6 in SK-N-BE2 and CHP-212 cells (Fig. 5M). Similarly, overexpression of TRIM59 increased the ubiquitination level of IRF6 in HEK293T cells, suggesting that IRF6 is subjected to ubiquitin-mediated degradation (Fig. 5N). To ascertain whether TRIM59 directly ubiquitinates IRF6, we performed an in vitro ubiquitination assay. As illustrated in Fig. 5O, polyubiquitin bands were detected when purified IRF6 was incubated with ubiquitin, UBA1/UBE1, UBE2D1, ATP, and TRIM59. Collectively, these results demonstrate that TRIM59 facilitated IRF6 ubiquitination and degradation through the ubiquitin‒proteasome pathway.

### IRF6 overexpression reverses TRIM59 glycolysis-mediated cell proliferation

**Fig. 6:**
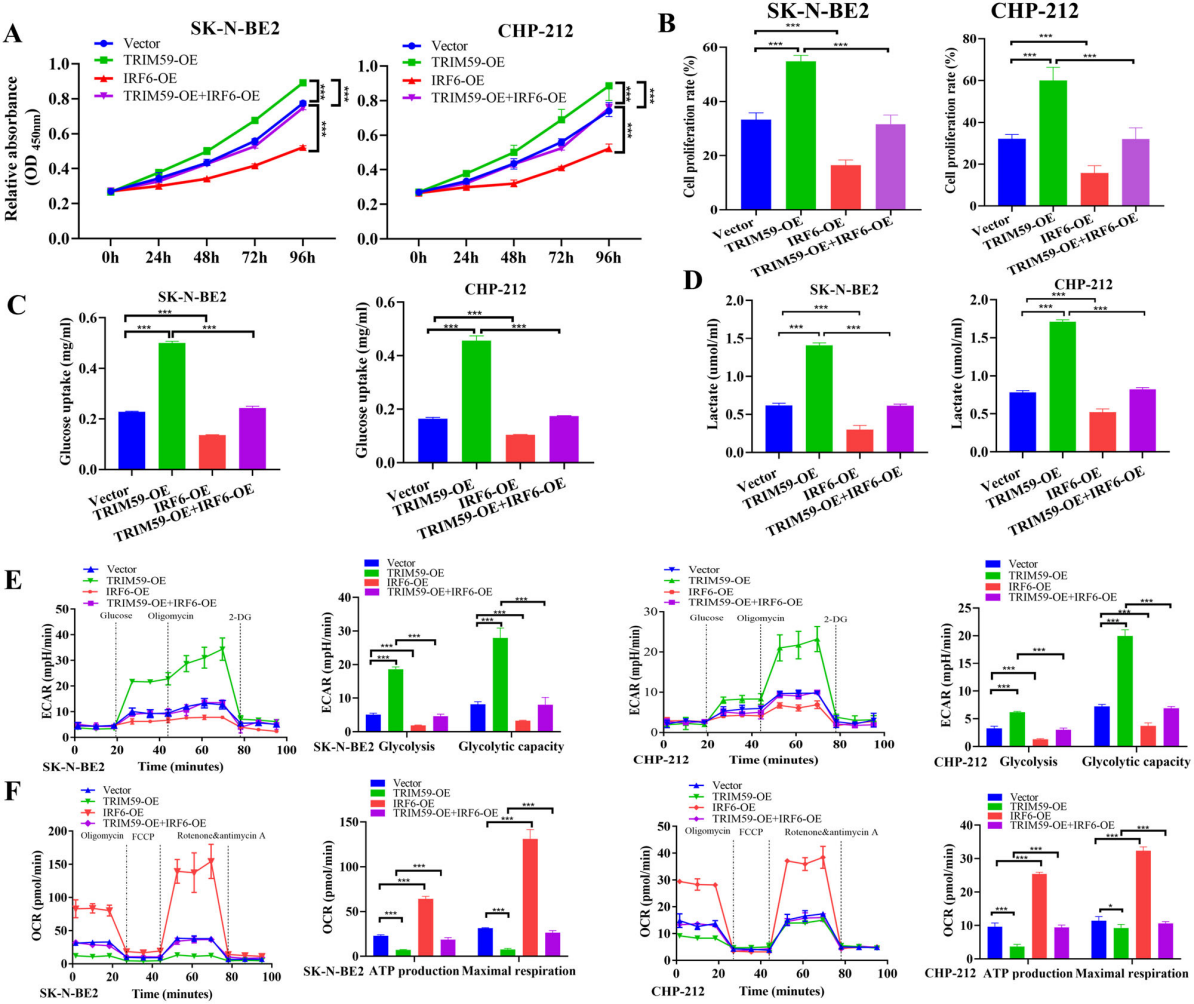
IRF6 reverses the promoting effect of TRIM59 on glycolysis-mediated tumor cell proliferation. SK-N-BE2 and CHP-212 cells were transiently cotransfected with Flag-IRF6 and the corresponding empty vector plus TRIM59 or the corresponding control plasmid and were then used for functional experiments. Cell viability (**A**) and proliferation (**B**) were evaluated by CCK-8 and EdU assays in SK-N-BE2 and CHP-212 cells, and simultaneously, glucose uptake (**C**), lactate (**D**), the ECAR (**E**), and the OCR (**F**) were evaluated. The data in **A**–**F** are presented as the means ± SDs of three independent experiments, and the *p* values were determined by two-tailed Student’s *t* test.

Based on the mechanism of IRF6 regulation by TRIM59-mediated ubiquitination, we assessed the functional phenotype of neuroblastoma cells after altering the expression levels of TRIM59 and IRF6. Therefore, we co-transfected SK-N-BE2 and CHP-212 cells with TRIM59 and Flag-IRF6 plasmids or the corresponding empty vectors for subsequent experiments. The results showed that TRIM59 inhibited the expression level of IRF6, an effect reversed by ectopic IRF6 expression (Supplementary Fig. 5A). The functional results revealed that the overexpression of IRF6 rescued the promoting effects of TRIM59 overexpression on cell viability (Fig. 6A) and proliferation rates in SK-N-BE2 and CHP-212 cells (Fig. 6B, Supplementary Fig. 5B).

Moreover, TRIM59 overexpression increased glucose uptake and lactate production, and these increases were reversed by ectopic IRF6 expression in SK-N-BE2 and CHP-212 cells (Fig. 6C, D). Similarly, the effects of TRIM59 overexpression on promoting glycolysis and glycolytic capacity were reversed by IRF6 overexpression in both SK-N-BE2 and CHP-212 cells (Fig. 6E), while the opposite was observed in the OCR assay (Fig. 6F). Taken together, these findings suggest that IRF6 is modulated by the E3 ligase TRIM59, and IRF6 overexpression counteracted TRIM59-mediated glycolysis-induced cell proliferation.

### Potential anticancer role of IRF6-PGM1 in neuroblastoma

**Fig. 7:**
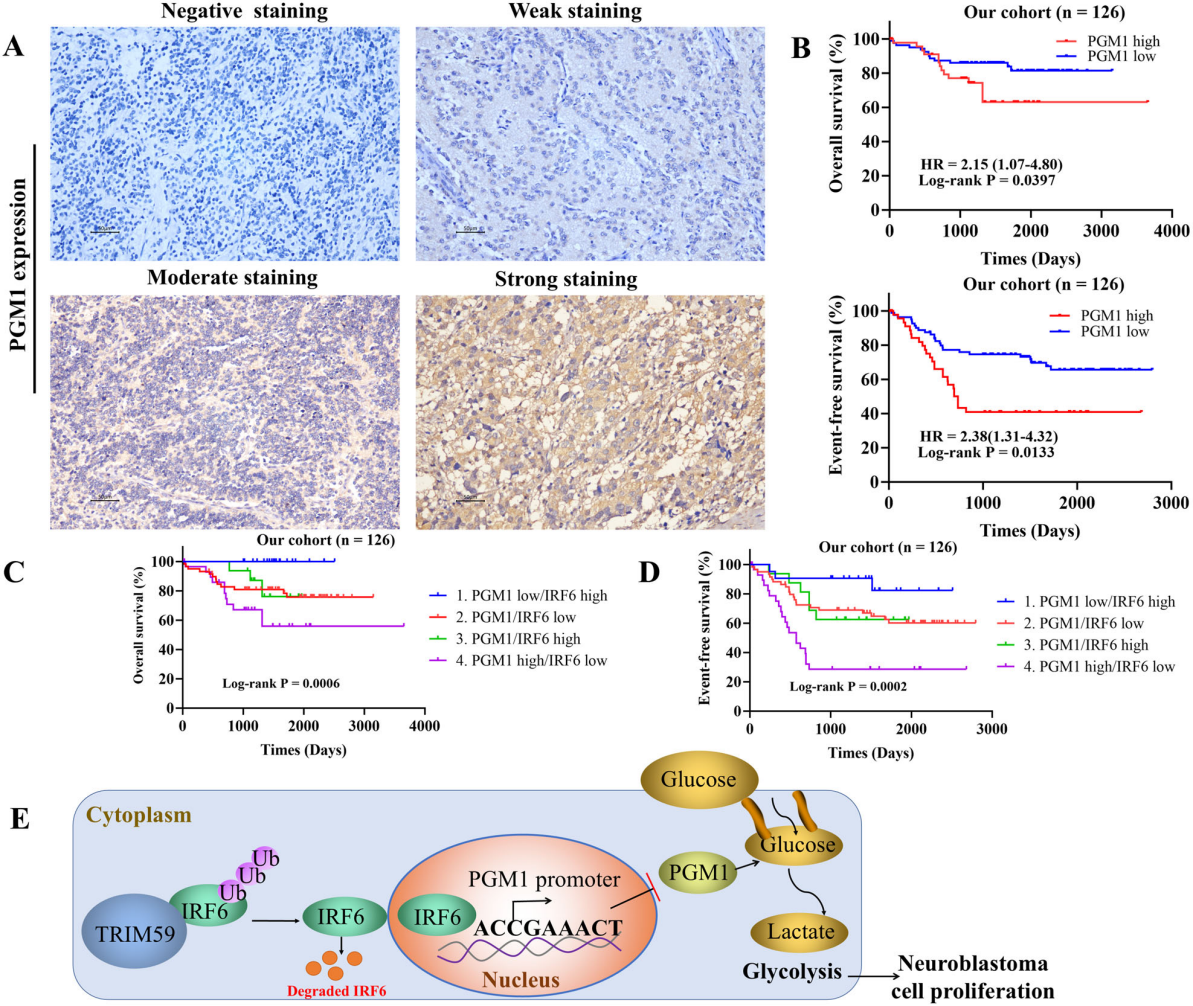
IRF6-PGM1 expression predicts neuroblastoma patient survival. **A** Representative images of PGM1 IHC staining in our dataset (*n* = 126). Scale bar, 50 μm. **B** Kaplan–Meier analysis of overall survival and event-free survival based on PGM1 expression in 126 patients with neuroblastoma. **C**, **D** Kaplan–Meier analysis of overall survival and event-free survival based on PGM1/IRF6 integrated expression in 126 patients with neuroblastoma. **E** A proposed model for the regulatory landscape of the TRIM59-IRF6-PGM1 pathway in neuroblastoma. The data in **B**–**D**, the *p* values were determined by the log-rank test.

Given the significant roles of IRF6 and PGM1 in glycolysis regulation and neuroblastoma progression, we analyzed their impact on patient survival in our cohort. The results revealed the presence of PGM1 in the cytoplasm, as determined by IHC staining analysis (Fig. 7A), and the high level of PGM1 expression was associated with poor overall and event-free survival in neuroblastoma patients (Fig. 7B). Notably, neuroblastoma patients with PGM1 high/IRF6 low integrated expression had the worst overall and event-free survival (Fig. 7C, D). In summary, our findings reveal the potential tumor-suppressing effects of the IRF6-PGM1 pathway in neuroblastoma.

## Discussion

In this study, we first revealed that IRF6 acts as a potent inhibitor of tumor cell growth by targeting PGM1 transcription and modulating the cellular glycolytic process in neuroblastoma. Moreover, IRF6 expression was downregulated due to an increased level of ubiquitination mediated by the E3 ligase TRIM59, which correlated with unfavorable outcomes in neuroblastoma patients. Clinically, neuroblastoma patients with PGM1 high/IRF6 low integrated expression had the worst overall and event-free survival, emphasizing the crucial role of the IRF6-PGM1 pathway in glycolysis-dependent tumorigenesis. This novel axis may be useful for developing new therapeutic strategies for patients, especially those at high risk.

Increasing evidence indicates that cancer cells preferentially utilize glycolysis to produce glucose-dependent ATP and glycolytic intermediates necessary for rapid growth, even with sufficient oxygen, which affects the assessment of the prognosis and therapeutic effect of cancers [22, 23]. Hence, the crucial correlation between tumorigenesis and glycolysis has been increasingly acknowledged. IRF6 is known to regulate apoptosis, cell cycle arrest, and epidermal differentiation [24, 25]. However, few studies have examined the role of IRF6 in the association between tumorigenesis and glycolytic reprogramming. Lu et al. recently reported that IRF6 is downregulated in glioma, with the Lin28A/SNHG14/IRF6 axis reprogramming glucose metabolism and stimulating tumorigenesis in glioma cells [13]. In our study, IRF6 was also found to be decreased in neuroblastoma, with its overexpression inhibiting glycolysis-mediated tumor cell growth. To the best of our knowledge, this is the first report on the decreased expression of IRF6 in neuroblastoma cells, and we demonstrated that IRF6 overexpression suppresses glycolysis-mediated tumor cell proliferation.

Our study revealed that PGM1 serves as a target for IRF6-mediated glycolysis and tumorigenesis in neuroblastoma cells. PGM1 initiates glycogenesis by catalyzing the reversible conversion between glucose 1-phosphate and glucose 6-phosphate and is involved in both the breakdown and synthesis of glycogen [26, 27]. Yang et al. demonstrated that PGM1 is downregulated in hepatocellular carcinoma, accompanied by increased levels of glycolysis, thereby promoting cell proliferation and tumor development [28]. In another study, it was shown that elevated levels of PGM1 in lung cancer tissues through AMPK activation under glucose-deprived conditions correlated with poor survival in those patients [29]. These findings suggest that PGM1 plays dual anti- and pro-cancer roles in multiple cancers. In our study, we found that IRF6 directly interacted with the PGM1 promoter, inhibiting its transcription; subsequently, the downregulation of PGM1 inhibited glycolysis-mediated tumor cell proliferation in neuroblastoma, highlighting the role of PGM1 in facilitating the progression of neuroblastoma.

Over the past decade, research has increasingly highlighted the impact of ubiquitination on regulating the stability of critical players in different aspects of metabolism [30]. TRIM proteins, which possess a tripartite motif and a RING finger domain, typically act as E3 ubiquitin ligases and are involved in multiple cellular processes, including cell proliferation, transcriptional regulation, and cancer progression [31–33]. Notably, extensive research has shown that TRIM59 serves as an oncogene in multiple types of human cancers, including ovarian, gastric, pancreatic, and non-small cell lung cancers. For example, TRIM59 interacts with the P53 tumor suppressor, leading to its ubiquitination and degradation in gastric cancer, consequently encouraging expansion, movement, and xenograft tumor growth [34]. A recent study highlighted the considerable upregulation of TRIM59 in recurrent bladder cancer, involving the F-actin/ITGB8/TRIM59/AKT/mTOR/glycolysis pathways [35]. Another study revealed that TRIM59 enhances ubiquitination of MKP3, with ectopic expression of MKP3 counteracting the promoting effect of TRIM59 on glycolysis [36]. In our study, we found that TRIM59 increased the ubiquitination level of IRF6 and reduced its protein level. Therefore, IRF6 overexpression reversed the ability of TRIM59 to promote glycolysis-dependent tumor cell growth. This mechanism underscores TRIM59’s role as a significant oncogene in our study. Collectively, these findings, along with our results, suggest that TRIM59 performs numerous complex functions, including glycolysis, which is crucial for cancer progression.

As described above, IRF6 has been identified as a prognostic marker in several cancers, but its prognostic value in neuroblastoma remains unknown. Our results revealed that decreased IRF6 expression is associated with an inferior prognosis in neuroblastoma patients, providing a predictive index for curative effects on neuroblastoma. Figure 7E displays the working model: IRF6 binds to the PGM1 promoter and transcriptionally inhibits PGM1 expression. Simultaneously, TRIM59 increased the ubiquitination level of IRF6 and caused its degradation. Consequently, IRF6 overexpression inhibits glycolysis and eventually represses tumor cell proliferation in neuroblastoma. Importantly, PGM1 may reverse the inhibitory effect of IRF6 on glycolysis-mediated tumor cell proliferation.

In conclusion, our study offers the first comprehensive evidence that the TRIM59-IRF6-PGM1 pathway plays a crucial role in glycolysis-induced tumorigenesis in neuroblastoma cells and may serve as a target for neuroblastoma therapy and prognosis.

## Supplementary information

Supplementary File

## Data Availability

The data used in this study are described in the methods section. All other data supporting the findings of this study are available upon request from the corresponding author or within the article and its supplementary data files.
