# Supplementary material for: Degradation of IRF6 by TRIM59 in tumor cells triggers PGM1-mediated glycolysis to regulate cell proliferation in neuroblastoma
Source: Cell Death Dis. 2025 Aug 12;16(1):613. doi: 10.1038/s41419-025-07932-2 (PMC12343910; doi:10.1038/s41419-025-07932-2)
Supplement: Supplementary file 1 — Supplementary File [file 41419_2025_7932_MOESM1_ESM.docx]

**Supplementary Materials**

**Degradation of IRF6 by TRIM59 in tumor cells triggers PGM1-mediated glycolysis to regulate cell proliferation in neuroblastoma**

**Zeng et al.**


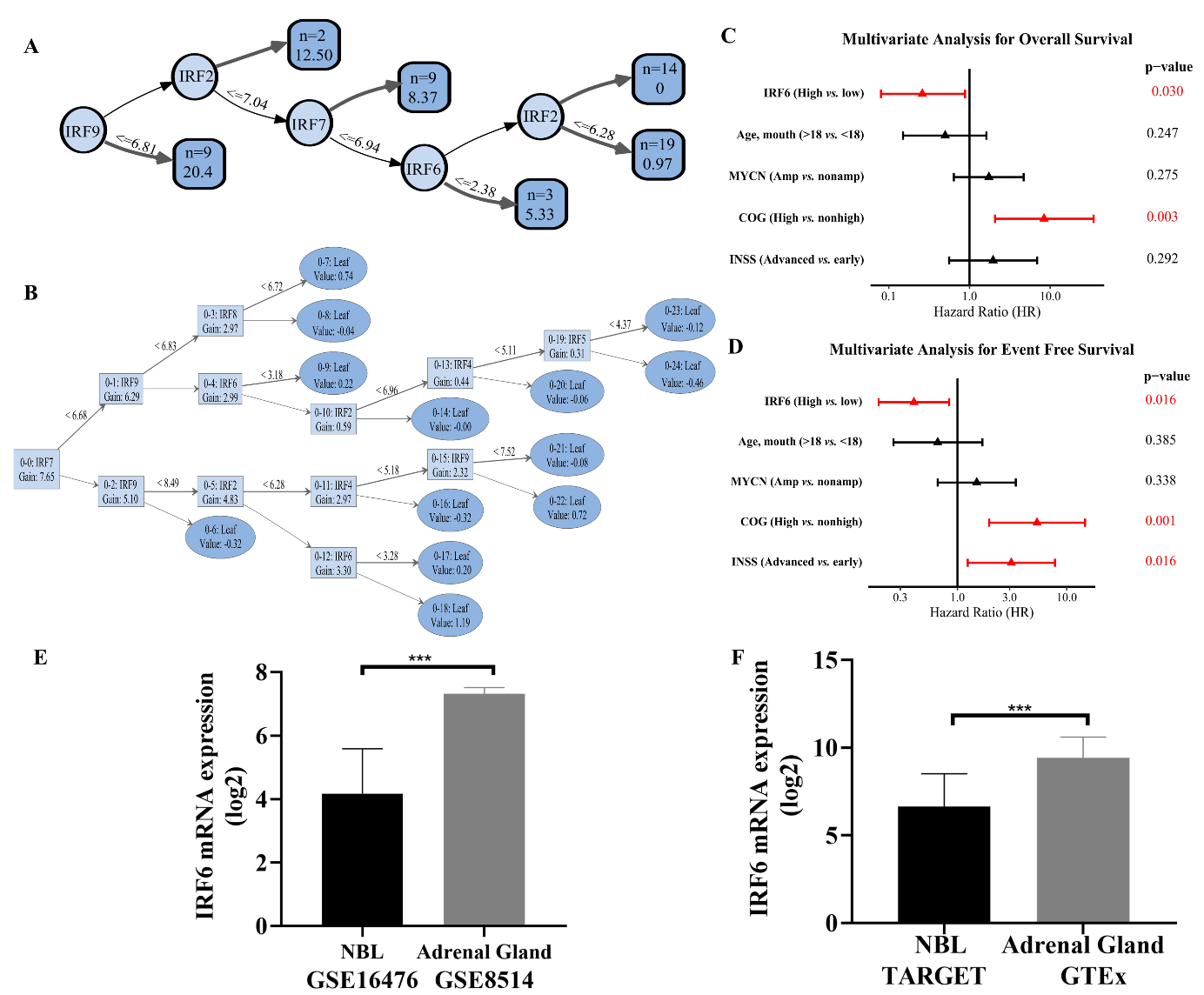


**Supplementary Fig. 1** **The screening strategy for IRF6, and its expression differences in neuroblastoma tissues compared to normal tissues.** (**A-B**) The GSE16476 dataset was used in the screening analysis of IRF1-9 contributing to the survival risk in neuroblastoma patients according to the random forest (**A**) and XGBoost algorithms (**B**), respectively. (**C-D**) Forest plots showing the significance of risk factors, including IRF6 and clinical features, to overall survival (**C**) and event-free survival (**D**) in our NBL cohort (n = 126). (**E-F**) Expression of IRF6 in one NBL cohort (GSE16476, n = 88) and one adrenal gland cohort (n = 5), another NBL cohort (TARGET, n = 153) and adrenal gland cohort from GTEx dataset (n = 125). The y-axis shows the log2 normalized expression values. **Abbreviations:** INSS, the International Neuroblastoma Staging System; COG, Children’s Oncology Group; NBL, neuroblastoma.


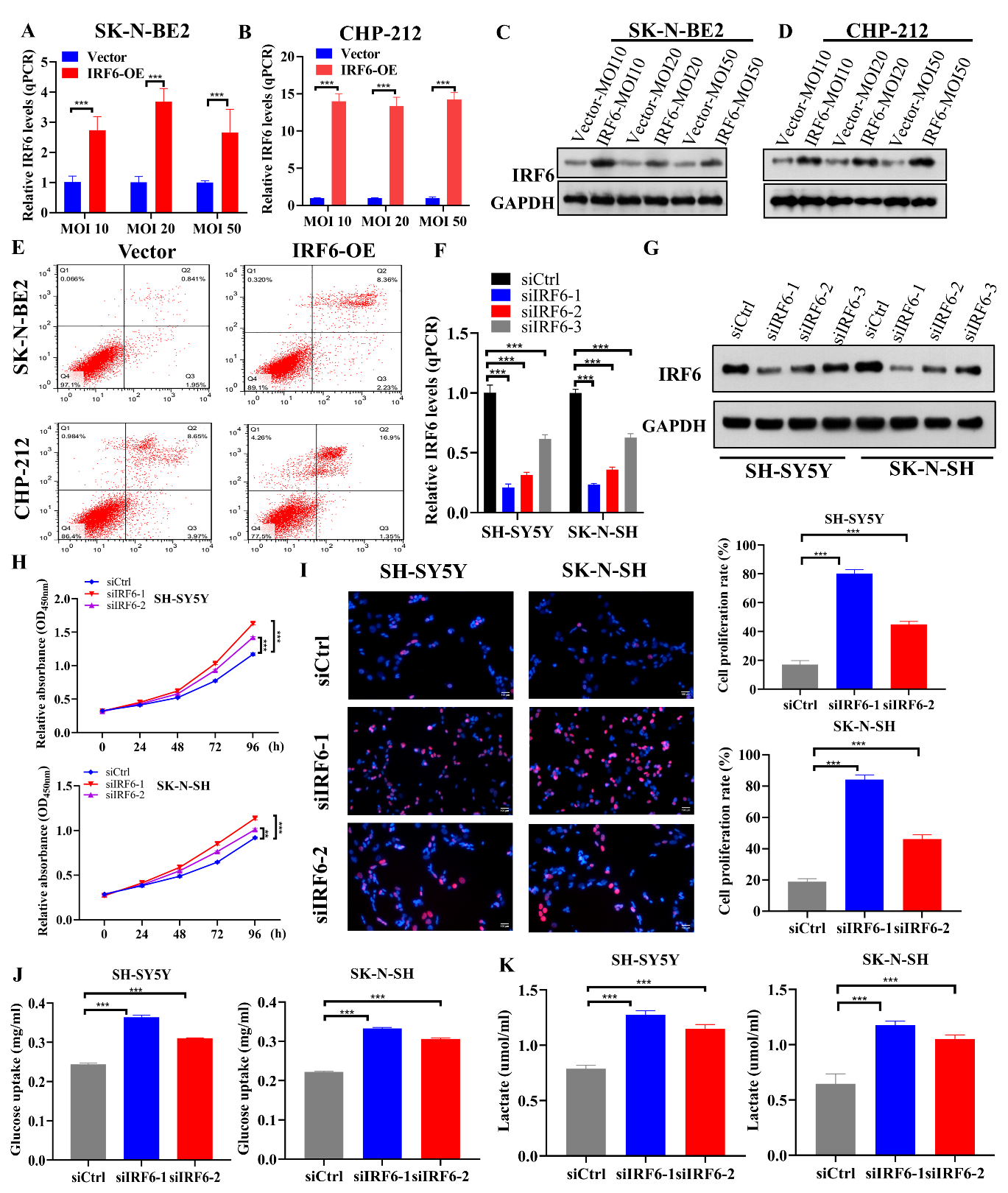


**Supplementary Fig. 2 IRF6 inhibits cell proliferation, apoptosis, glucose uptake, and lactate production in NBL cells.** (**A-B**) RT‒qPCR analyses of IRF6 expression in SK-N-BE2 and CHP-212 cells after transfection with the viral supernatant containing Flag-IRF6 or the corresponding empty plasmids with MOI10, MOI20, and MOI50, respectively. (**C-D**) Western blot analyses of IRF6 expression in SK-N-BE2 and CHP-212 cells after transfection with the viral supernatant containing Flag-IRF6 or the corresponding empty plasmids with MOI10, MOI20, and MOI50, respectively. The indicated cells treated with the MOI20 were used for further experiments. (**E**) After SK-N-BE2 and CHP-212 cells were transfected with Flag-IRF6 or the corresponding empty vector, cell apoptosis was evaluated by flow cytometry. RT-qPCR (**F**) and western blot (**G**) analyses of IRF6 expression in SH-SY5Y and SK-N-SH cells after transfection with siRNA plasmids, such as siCtrl, siIRF6-1, siIRF6-2, and siIRF6-3. After SH-SY5Y and SK-N-SH cells were transfected with siIRF6 or the corresponding siCtrl, cell proliferation viability was evaluated using a CCK-8 assay (**H**) and an EdU assay (**I**), glucose uptake (**J**) and lactate production (**K**) were also evaluated. The data in **A-B, F, H-K** are presented as the means ± SDs, and the *p* values were determined by two-tailed Student’s *t* test. **Abbreviations:** MOI, multiplicity of infection; OE, overexpression; NBL, neuroblastoma.


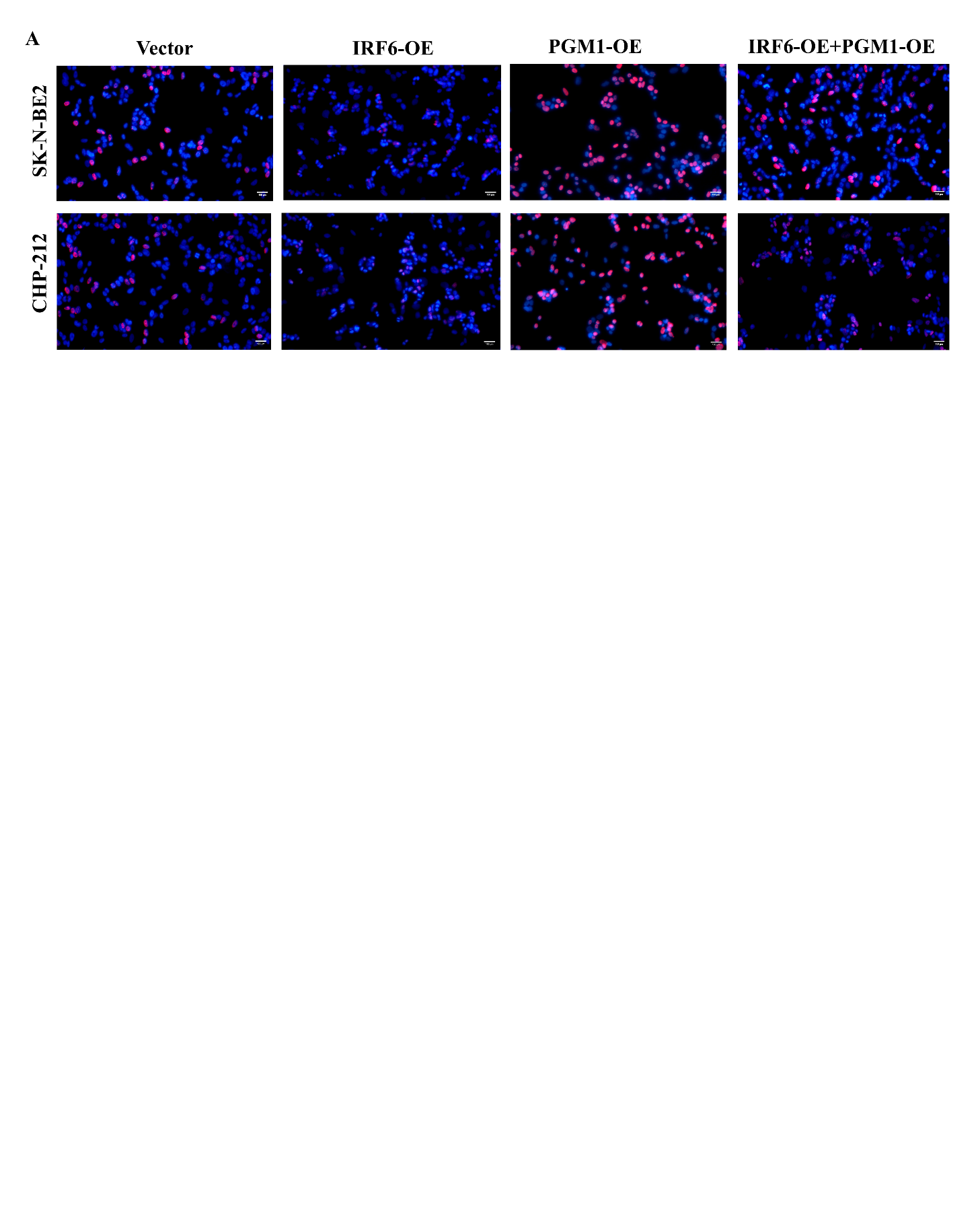


**Supplementary Fig. 3 Effect of IRF6 expression on cell proliferation in neuroblastoma cells.** SK-N-BE2 and CHP-212 cells were transiently cotransfected with Flag-IRF6 or the corresponding empty vector plus PGM1 or the corresponding control plasmid and were then used for functional experiments. Representative images for cell proliferation rate with EdU assay (**A**). **Abbreviations**: OE, overexpression.


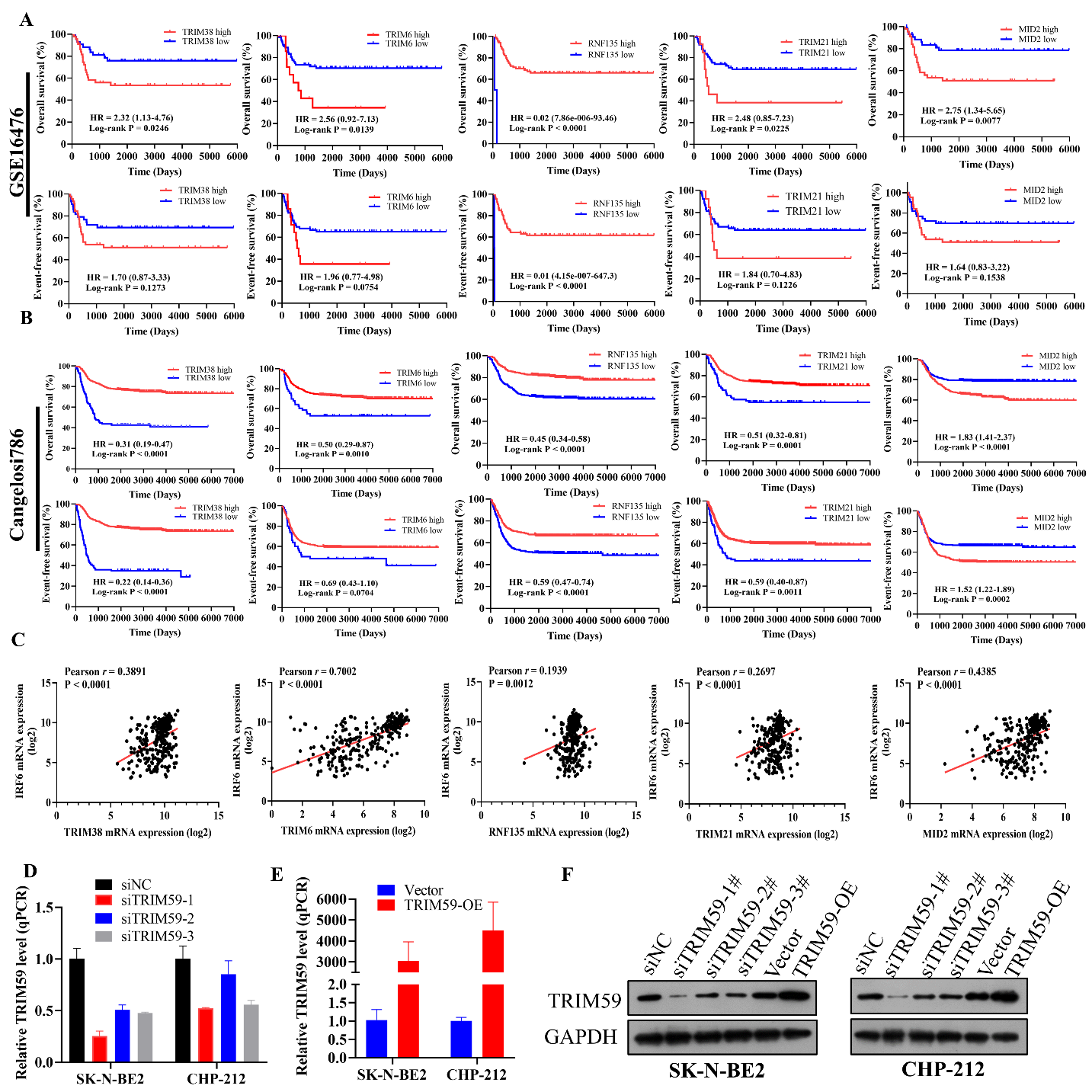


**Supplementary Fig. 4 Correlation between the other five E3 ligases and clinical outcome in neuroblastoma patients.** (**A-B**) Kaplan–Meier analysis of overall survival and event-free survival based on the expression of the other five E3 ligases, such as TRIM38, TRIM6, RNF135, TRIM21, and MID2 in the GSE16476 (**A**) and Cangelosi786 datasets (**B**) with neuroblastoma. (**C**) The positive correlation of the indicated E3 ligases and IRF6 expression is assessed according to the expressions of the indicated E3 ligases and IRF6 from the GTEx and TARGET 153 datasets. (**D-E**) RT‒qPCR analyses of TRIM59 expression in SK-N-BE2 and CHP-212 cells after transfection with siTRIM59 or TRIM59 or the corresponding empty vector. (**F**) Western blot analyses of TRIM59 expression in SK-N-BE2 and CHP-212 cells after transfection with siTRIM59 or TRIM59 or the corresponding empty vector. The data in **D-E** are presented as the means ± SDs, and the *p* values were determined by two-tailed Student’s *t* test.


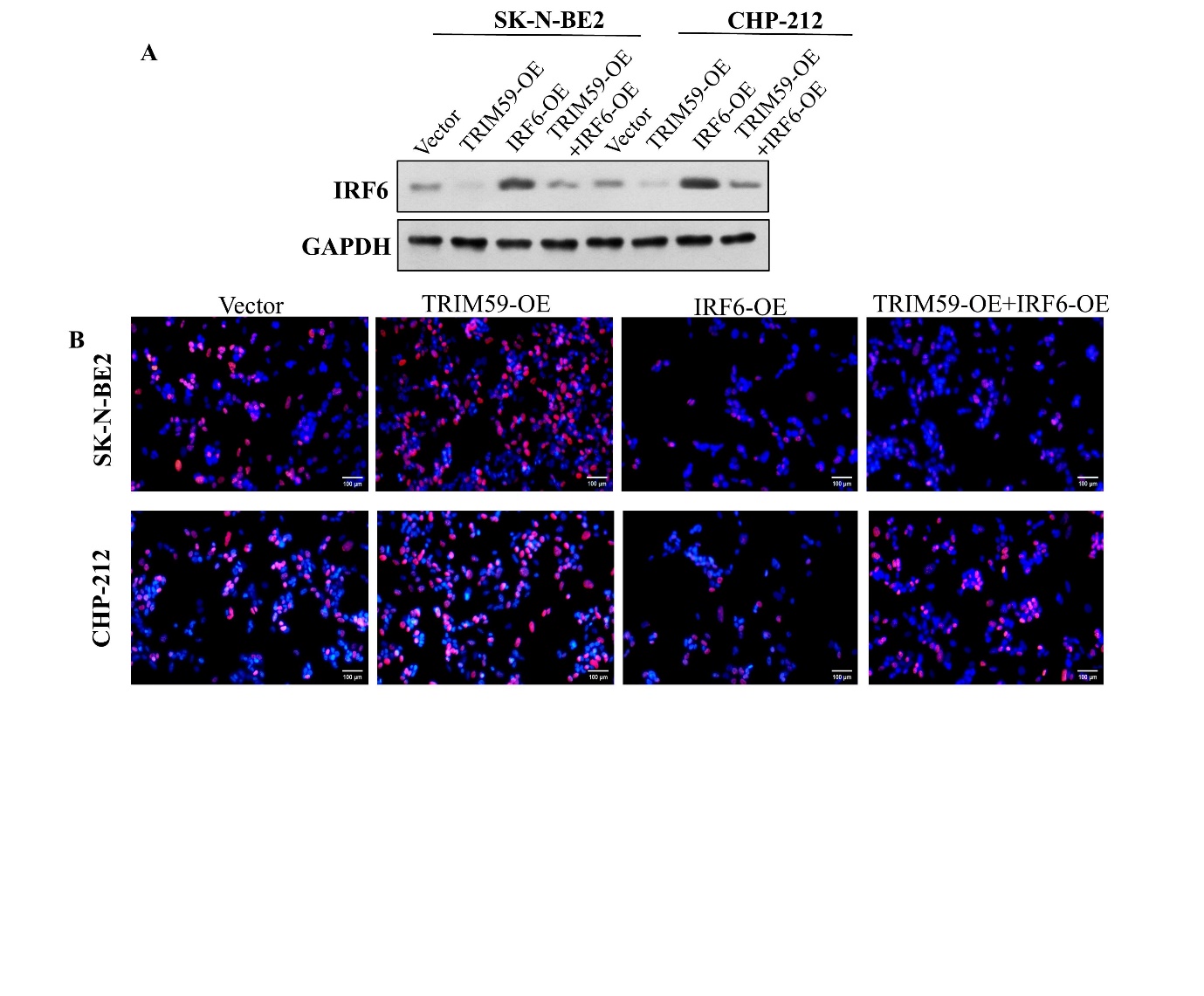


**Supplementary Fig. 5 The verification of cell transfection and the effect of TRIM59 expression on cell proliferation in neuroblastoma cells.** Western blot analyses show IRF6 reversed the inhibitory effect of TRIM59 on IRF6 expression in SK-N-BE2 and CHP-212 cells (**A**). SK-N-BE2 and CHP-212 cells were transiently cotransfected with Flag-IRF6 or the corresponding empty vector plus TRIM59 or the corresponding control plasmid and were then used for functional experiments. Representative images for cell proliferation rate with EdU assay (**B**).


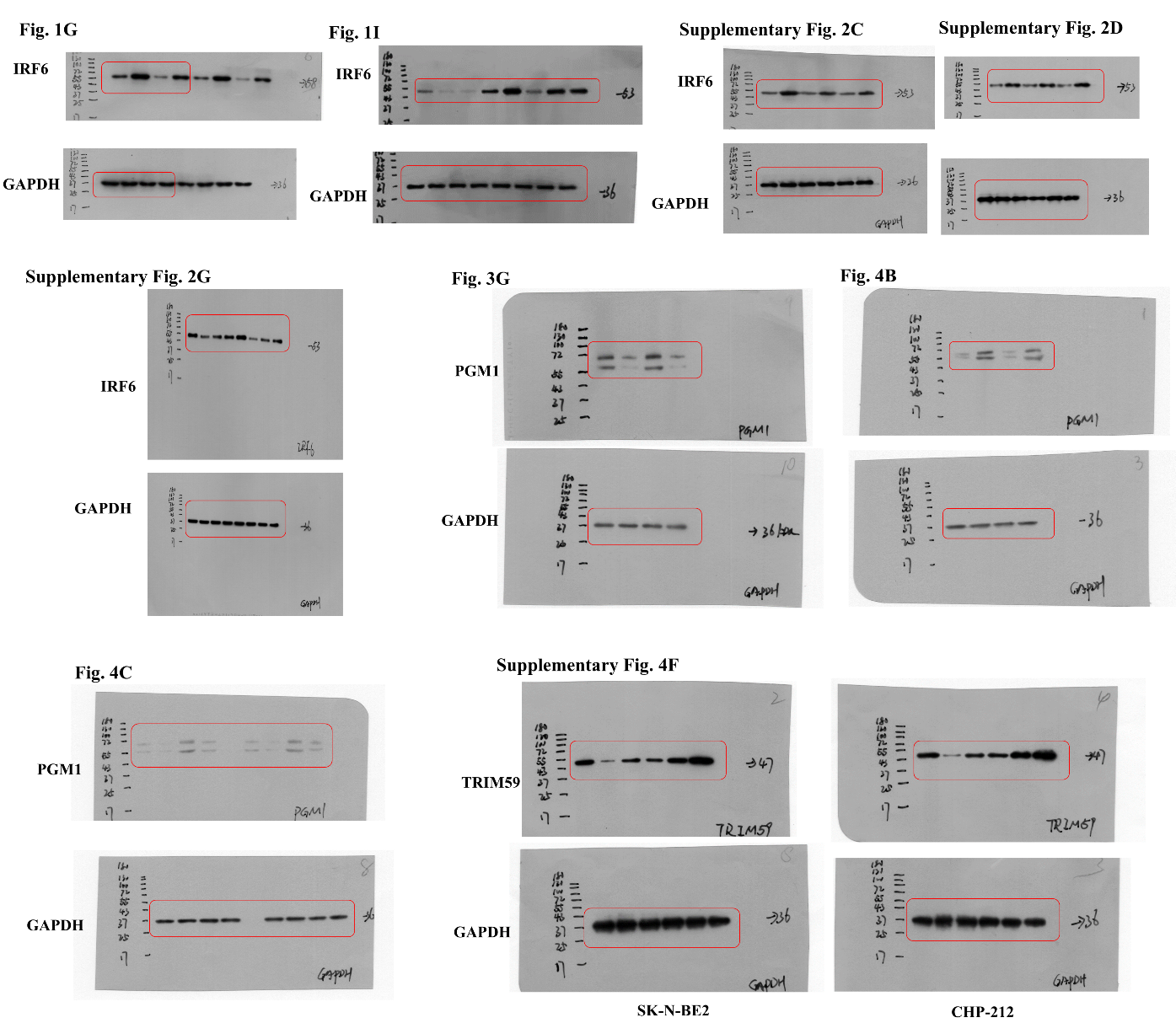


**Supplementary Fig. 6** Unprocessed immunoblots for indicated Figures panels.


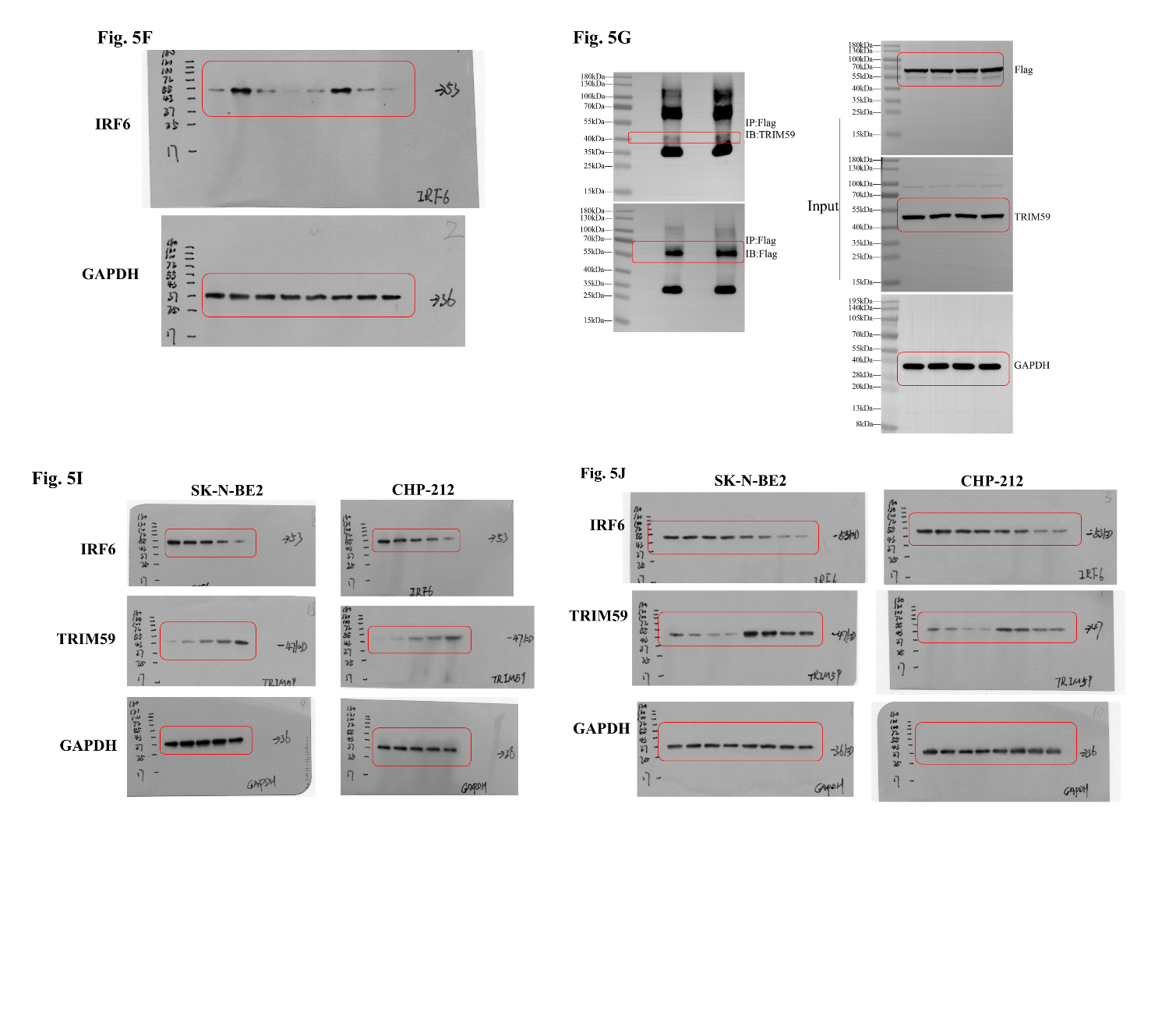


**Supplementary Fig. 6 (cont’d)** Unprocessed immunoblots for indicated Figures panels.


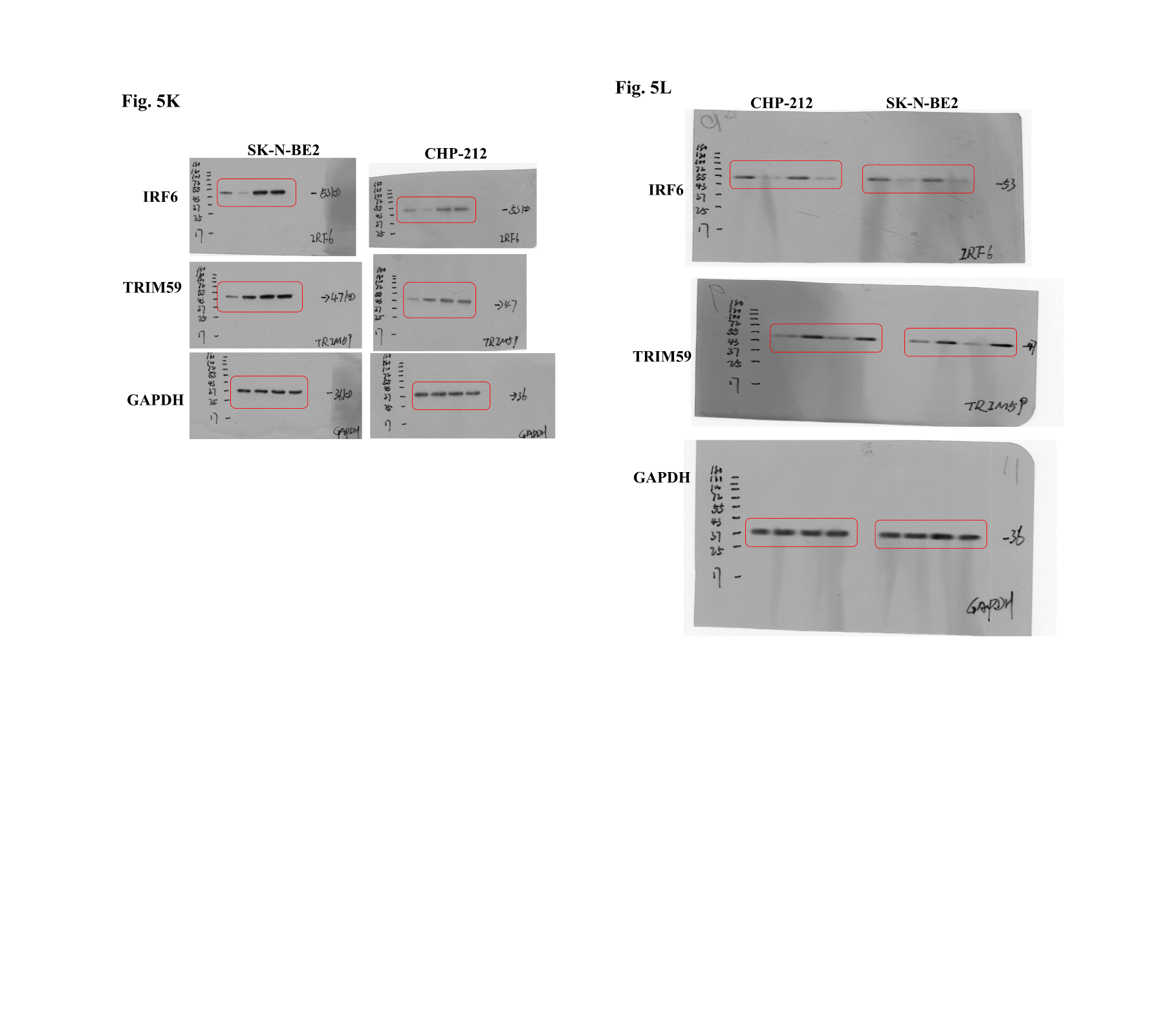


**Supplementary Fig. 6 (cont’d)** Unprocessed immunoblots for indicated Figures panels.


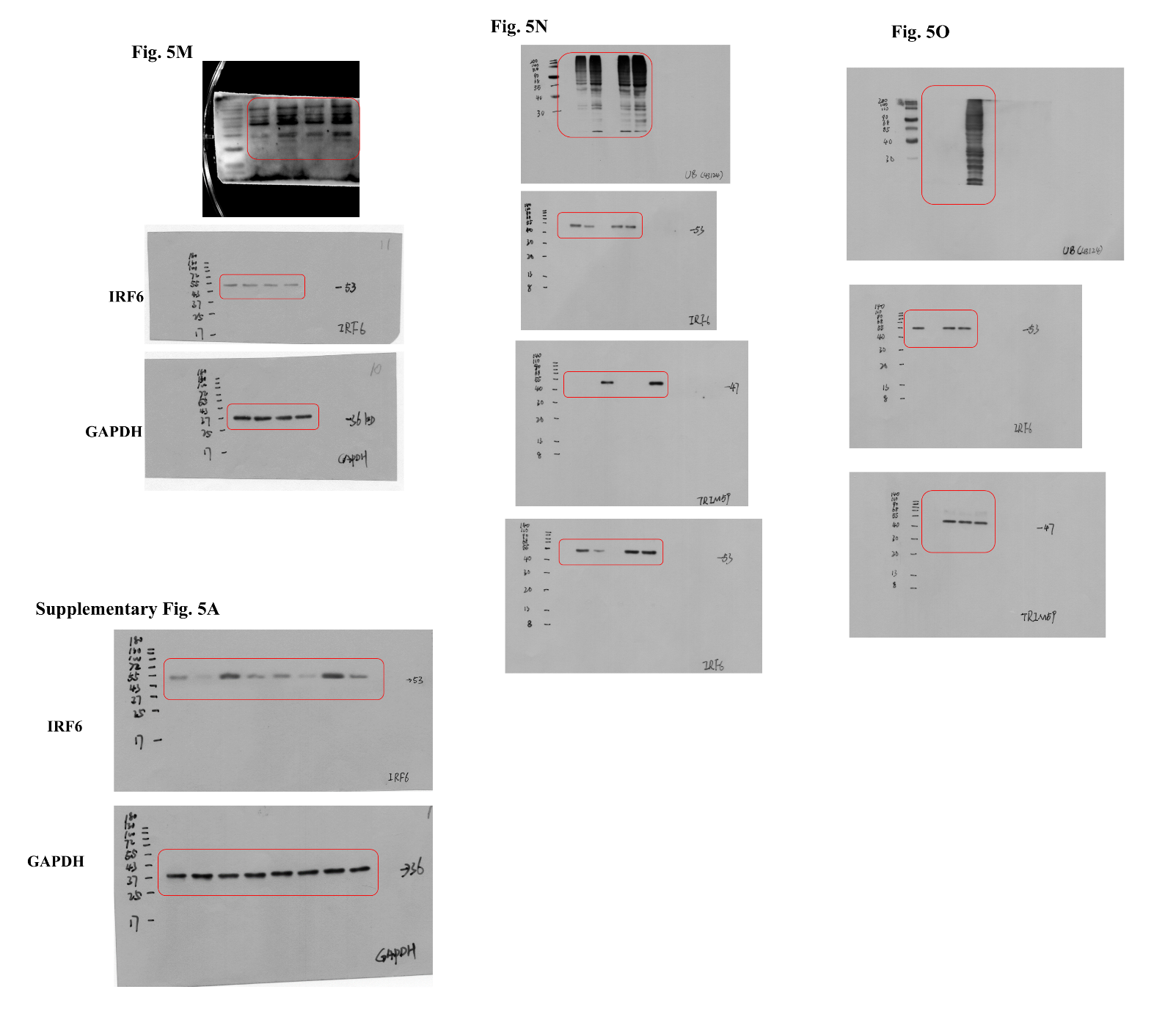


**Supplementary Fig. 6 (cont’d)** Unprocessed immunoblots for indicated Figures panels.

| **Supplementary Table 1.** Primers for RT-qPCR, siRNAs, and vector construction | |
| --- | --- |
| Name | Sequences (5'-3') |
| **Primers for RT-qPCR** |  |
| IRF6 F | GAGCTCTTTGGTCCCGTCA |
| IRF6 R | AATGGCATGACCGCTGACC |
| TRIM59 F | TTCCTAACGGCTCTCTGTGATG |
| TRIM59 R | TTAATTCAAGCTGCTGCTCTCG |
| PGM1 F | ATCCGTGAGAAAGATGGACTGT |
| PGM1 R | GTGAAGAAATTCCGGCCATACT |
| β-catenin F | CACCATTGGCAATGAGCGGTTC |
| β-catenin R | AGGTCTTTGCGGATGTCCACGT |
| **siRNA sequences** |  |
| siTRIM59-1 F | ACAUUACAGGCAACCAUUAAAdTdT |
| siTRIM59-1 R | UUUAAUGGUUGCCUGUAAUGUdTdT |
| siTRIM59-2 F | CCAACUGGCAUUGAAUCUUUAdTdT |
| siTRIM59-2 R | UAAAGAUUCAAUGCCAGUUGGdTdT |
| siTRIM59-3 F | AGGAAGCUGUUCUCCAGUAUUdTdT |
| siTRIM59-3 R | AAUACUGGAGAACAGCUUCCUdTdT |
| siIRF6-1 F | CUGAGCAUAUUACCAAUGAdTdT |
| siIRF6-1 R | UCAUUGGUAAUAUGCUCAGdTdT |
| siIRF6-2 F | CUCGGAUGAUCUACGAGAUdTdT |
| siIRF6-2 R | AUCUCGUAGAUCAUCCGAGdTdT |
| siIRF6-3 F | GCACCUAUACAGCCCUUCUdTdT |
| siIRF6-3 R | AGAAGGGCUGUAUAGGUGCdTdT |
| **Primers for plasmid constructs (Vector plasmid: 3FLAG-EF1a-firefly-Luciferase-SV40)** | |
| IRF6 F | AGGTCGACTCTAGAGGATCCCGCCACCATGGCCCTCCACCCCCGCAG |
| IRF6 R | TCCTTGTAGTCCATGGATCCCTGGGGAGGCAGGGCAGGGGGCAG |
| **Primers for plasmid constructs (Vector plasmid: pcDNA3.1)** | |
| TRIM59 F | ACTAAAGCTTATGCACAATTTTGAGGAAGA |
| TRIM59 R | ACTTGGATCCTCAATGGGAAACTATTTTCCAC |
| PGM1 F | ACTAGGATCCGCCACCATGGTGAAGATCGTGACAGT |
| PGM1 R | ACTTGAATTCTTAGGTGATGACAGTGGGT |
| Primers for plasmid constructs (Vector plasmid: **pcDNA3.1-TRIM59**) | |
| HA F | CAAGCTGGCTAGCGTTTAAACTTAAGCTTGCCACCATGTACCCATA TGACGTTCCAG |
| HA R | TACTATAACATATGGGACAAGTTAACTCTTCCTCAAAATTGTGAGC GTAGTCAGGTACATC |
| Primers for plasmid constructs (Vector plasmid: **pET28a**) | |
| Myc-IRF6 F | ACTATCTAGAAATAATTTTGTTTAACTTTAAGAAGGAGATAT  ACCATGGAGCAGAAACTCATCTCAG |
| Myc-IRF6 R | ACTACTCGAGTTACTGGGGAGGCAGGGCAGG |
| **Primers for ChIP-qPCR** | |
| PGM1-primer 1-F | AATTGTTACAACTTCTGGGGTGA |
| PGM1-primer 1-R | TCACGGGCCCAAATTCAGATCTCA |
| PGM1-primer 2-F | ACTTATTAGCACTGTGGTAGGAGC |
| PGM1-primer 2-R | AGAGAATTGAGGATTGGATGGTG |
| PGM1-primer 3-F | ACTAGCAGTCCAGGGTTCCGT |
| PGM1-primer 3-R | GAGGCGAACAGACCCATAAGA |
| PGM1-primer 4-F | ACCACTGGAAATCATTCTACCCT |
| PGM1-primer 4-R | CCTTCGGAATCCTATCCACTGT |
| **Primers for dual-luciferase assay** | |
| PGM1-primer F | ACTACTCGAGATTATTTAATTCACAAGATTAC |
| PGM1-primer R | ACTTAAGCTTACCCATAAGAGTGCAGACTCT |
|  |  |

| **Supplementary Table 2.** List of antibodies used in this study | |  |  |
| --- | --- | --- | --- |
| Antibody | Company | Catalog No. | Dilution |
| **Western blot** |  |  |  |
| anti-IRF6, Rabbit Monoclonal | SAB | 49146 | 1:1000 |
| anti-IRF6, Mouse Monoclonal | Abcam | ab123880 | 1:1000 |
| anti-TRIM59, Rabbit Polyclonal | Abcam | ab166793 | 1:1000 |
| anti-PGM1, Rabbit Polyclonal | SAB | 39603 | 1:1000 |
| anti-GAPDH, HRP-conjugated mouse monoclonal | Kangchen | KC-5G5 | 1:10000 |
| **Immunohistochemistry** |  |  |  |
| anti-IRF6, Rabbit Monoclonal | SAB | 49146 | 1:300 |
| anti-Ki67, Rabbit Monoclonal | AccuPath | IR098:LBP2-Ki67 | ready-to-use |
| anti-PGM1, Rabbit Polyclonal | SAB | 39603 | 1:200 |
| **Co-IP** |  |  |  |
| anti-Flag-tag, Rabbit Monoclonal | CST | 14793 | 2 μg |
| anti-Ubiquitin (E4I2J) Rabbit Monoclonal | CST | 43124S | 2 μg |
| anti-IgG, Rabbit Polyclonal | Proteintech | 30000-0-AP | 2 μg |
| anti-IgG, Mouse Polyclonal | Proteintech | B900120 | 2 μg |
| anti-GST, Rabbit Monoclonal | Diagbio | Db11058 | 2 μg |
| anti-His, Mouse Monoclonal | Proteintech | 66005-1-Ig | 2 μg |
| anti-Myc, Rabbit Monoclonal | CST | 71D10 | 2 μg |
| Anti-HA, Mouse Monoclonal | MBL | M180-3 | 2 μg |
| **ChIP-qPCR** |  |  |  |
| anti-Flag-tag, Rabbit Monoclonal | CST | 14793 | 2 μg |
| anti-IgG, Rabbit Monoclonal | Bersinbio | Bes5001 | 2 μg |
|  |  |  |  |

| **Supplementary Table 3.** Correlation between IRF6 expression and clinicopathologic features in patients with neuroblastoma (n = 126) | | | |
| --- | --- | --- | --- |
| Variable | IRF6 expression | | *p* value |
|  | High (n, %) | Low (n, %) |  |
| **Age** |  |  |  |
| < 18 months | 21 (56.8) | 53 (59.6) | 0.772 |
| > 18 months | 16 (43.2) | 36 (40.4) |  |
| **Sex** |  |  |  |
| Female | 16 (43.2) | 45 (50.6) | 0.454 |
| Male | 21 (56.8) | 44 (49.4) |  |
| **Pathological grade** |  |  |  |
| Poor | 29 (80.6) | 74 (83.2) | 0.731 |
| Well | 7 (19.4) | 15 (16.8) |  |
| **MYCN status** |  |  |  |
| non-Amp | 36 (97.3) | 81 (91.0) | 0.212 |
| Amp | 1 (2.7) | 8 (9.0) |  |
| **COG** |  |  |  |
| non-High | 26 (70.3) | 56 (62.9) | 0.431 |
| High | 11 (29.7) | 33 (37.1) |  |
| **INSS** |  |  |  |
| Early | 14 (37.8) | 35 (39.3) | 0.876 |
| Advanced | 23 (62.2) | 54 (60.7) |  |
| **Overall survival** |  |  |  |
| Live | 34 (91.9) | 65 (73.0) | **0.019** |
| Dead | 3 (8.1) | 24 (27.0) |  |
| **Event-free survival** |  |  |  |
| No | 28 (75.7) | 46 (51.7) | **0.013** |
| Yes | 9 (24.3) | 43 (48.3) |  |
| **Abbreviations**: Amp, amplification; COG, children’s oncology group; INSS, international neuroblastoma staging system; IRF6, interferon regulatory factor 6. The *p* value was determined by χ2 or Fisher’s exact tests and bold values indicate *p* < 0.05. | | | |
|  |  |  |  |
